# A Fully‐Integrated Bayesian Approach for the Imputation and Analysis of Derived Outcome Variables With Missingness

**DOI:** 10.1002/sim.70383

**Published:** 2026-01-22

**Authors:** Harlan Campbell, Tim P. Morris, Paul Gustafson

**Affiliations:** ^1^ Department of Statistics University of British Columbia Vancouver Canada; ^2^ Health Economics and Outcomes Research Precision AQ Vancouver Canada; ^3^ MRC Clinical Trials Unit at UCL University College London London UK

**Keywords:** Bayesian inference, missingness, multiple imputation

## Abstract

Derived variables are variables that are constructed from one or more source variables through established mathematical operations or algorithms. For example, body mass index (BMI) is a derived variable constructed from two source variables: weight and height. When using a derived variable as the outcome in a statistical model, complications arise when some of the source variables have missing values. In this paper, we propose how one can define a single fully integrated Bayesian model to simultaneously impute missing values and sample from the posterior. We compare our proposed method with alternative approaches that rely on multiple imputation (MI), with examples including an analysis to estimate the risk of microcephaly (a derived variable based on sex, gestational age, and head circumference at birth) in newborns exposed to the ZIKA virus.

## 1 Introduction

Derived outcome variables are study outcomes that are constructed from one or more measured source variables through established mathematical operations or algorithms. Examples of commonly used derived outcome variables include body mass index (BMI), constructed from weight and height (e.g., [1, 2]), and the 36‐Item Short Form Health Survey Physical Component Summary score (SF‐36 PCS), constructed from a combination of responses measured on the 36‐Item Short Form Survey (SF‐36) (e.g., [3]).

When using a derived outcome variable, a complication may arise when some of the measured “source” variables required to derive it have missing values. Missingness in source variables will often lead to missingness in derived variables (e.g., if weight is missing for certain study participants, their BMI cannot be derived). However, this is not quite the same as having completely missing information on the derived outcome variable. In some instances, one might have some information. For example, if a single question on the SF‐36 is left unanswered, one might still be able to infer, at least approximately, a participant's SF‐36 PCS based on their answers to the other 35 questions. More clearly, if a composite outcome is defined to be 1 if either component is 1, then observing that one component equals 1 means the composite is known whether or not the other component is measured [4].

The data that initially motivated this work consist of measurements taken from newborns of mothers infected with the ZIKA virus [5, 6]. In order to determine if the newborns have microcephaly (a derived outcome variable), a calculation based on three source variables (sex, gestational age, and head circumference) is required. The source variables are often subject to missingness, and we were curious as to how one might be able to fit these data with an entirely Bayesian solution. The main advantage of a fully integrated Bayesian approach is that the joint posterior distribution will be estimated simultaneously for both unknown parameters and missing data, such that all sources of uncertainty are properly taken into account [7].

Besides the so‐called *complete‐case analysis*, in which observations with any missing values are discarded, the most popular statistical approach for handling missing data is multiple imputation (MI) [8, 9], and among the different varieties of MI, the multivariate imputation by chained equations (MICE) approach is the most widely used [10]. The basic idea of MI is to fill in missing values by repeated simulation from the posterior predictive distributions of the missing values, given assumptions about the missing data mechanism and a model, thereby preserving the uncertainty associated with the missingness. MICE does this for multivariate nonmonotone missing data variables by using a sequence of univariate conditional distributions (i.e., imputing variables one‐at‐a‐time).

When using MICE to impute missing values of derived variables, there are four general approaches:

1. imputation at the derived‐variable level (DVL), in which one imputes the derived variable directly, ignoring the source variables;
2. imputation at the source‐variable level (SVL), in which one imputes the source variables first and then computes the derived variable afterwards (also known as the *impute, then transform* approach);
3. the *just another variable* (JAV), approach, in which one imputes the source variables and derived variable together, with the source variables as covariates in the imputation model for the derived variable. To be clear, all variables are imputed separately, ignoring any deterministic relationships. Instead, multivariate normality between the derived variable and the source variables is often assumed (e.g., [11] and [12]). Notably, Seaman et al. (2012) [13] conclude that JAV should not be used to impute data for a logistic regression analysis as it performs “very badly” in this case; and
4. The *“on‐the‐fly” imputation*, where the transformation of the source variables into the derived variable is done “on‐the‐fly” within the imputation algorithm. (Note that Van Buuren (2018) [10] calls this “passive imputation”). The “on‐the‐fly” imputation consists of three steps. First, the source variables are imputed, then the derived variable is updated based on the known deterministic relationship with the source variables, and, third, all other variables are imputed conditional on this updated derived variable. When missingness only occurs in the derived variable and source variables (i.e., no other variables need imputing) and missingness in source variables necessarily implies missingness in the derived variable (e.g., any individual with unknown height or weight will necessarily have unknown BMI), on‐the‐fly imputation is entirely equivalent to SVL imputation.

The other approach we should mention, proposed by Bartlett et al. (2015) [14], is known as the Substantive Model Compatible‐Fully Conditional Specification (SMC‐FCS) imputation. While this approach ensures compatibility and allows for consistent estimation [15], it has only been applied to scenarios with derived *covariate* values. It is not immediately obvious how one could conceptually extend this approach to work with derived *outcome* variables, and current software implementations are limited [14, 16].

There may be challenges when imputing at the SVL if the derived variable is not a linear function of the source variables [13, 17, 18]. However, because DVL imputation effectively excludes information available in the source variables (which are possibly the best predictors of the missing derived‐variable values), SVL (or “on‐the‐fly”) imputation is thought to be a more efficient strategy, in the sense that it uses observed information where possible. Pan et al. (2020) [19] compare the performance of the DVL and SVL approaches and conclude that SVL imputation is indeed preferable, regardless of how the derived variable is obtained (“whether by simple arithmetic operations or by some highly specific algorithms”). This follows related work by Gottschall et al. (2012) [20] who arrived at a similar conclusion for handling missing values in questionnaire data (“researchers should adopt item‐level imputation whenever possible”).

Another reason to favor the SVL, “on‐the‐fly,” and JAV approaches over DVL imputation is that they make a less restrictive missing‐at‐random (MAR) assumption. MAR requires that the conditional probability of missingness depends only on data observed under that pattern [9]. Consider, for example, a derived outcome Y that is equal to the sum of two source variables, $Z_{1}$ and $Z_{2}$, such that $Y=Z_{1}+Z_{2}$. Missingness in Y can occur in three patterns: First, the $Z_{1}$ value is missing, second the $Z_{2}$ value is missing, third, both $Z_{1}$ and $Z_{2}$ values are missing; see Table 1. Because MAR is (i) defined at the *observation* level rather than at the variable level, and (ii) conditional on variables included in the analysis, we see that assuming MAR with DVL imputation requires that missingness in the derived variable Y be independent of the value of $Z_{1},Z_{2}$ under any pattern, see Table 1. For the SVL, “on‐the‐fly,” and JAV approaches, this is relaxed under patterns 1 and 2: missingness in $Z_{1}$ under pattern 1 need only be independent of $Z_{1}$, not $Z_{2}$, and missingness in $Z_{2}$ under pattern 2 need only be independent of $Z_{2}$, not $Z_{1}$.

**TABLE 1:** For each of the patterns, this table shows what the MAR assumption requires missingness be independent of. For the DVL imputation approach, missingness in the Y must be independent of the value of $Z_{1}$ and the value of $Z_{2}$, regardless of pattern.

Pattern	$A=Z_{1}$	$A=Z_{2}$	A=Y
	(source)	(source)	(derived)
	The MAR assumption requires that, under this pattern, missingness in A is independent of:		
1. ($Z_{1}$ Missing, $Z_{2}$ Observed) → Y Missing	$Z_{1}$	—	$Z_{1},Z_{2}$
2. ($Z_{1}$ Observed, $Z_{2}$ Missing) → Y Missing	—	$Z_{2}$	$Z_{1},Z_{2}$
3. ($Z_{1}$ Missing, $Z_{2}$ Missing) → Y Missing	$Z_{1},Z_{2}$	$Z_{1},Z_{2}$	$Z_{1},Z_{2}$

To conduct Bayesian inference with imputed data, Gelman et al. (2004) [21] outline a three‐step procedure (see also Zhou and Reiter (2010) [22]). The first step is to construct K imputed datasets, using MI. The second step is to simulate draws from the posterior distribution of the model parameters with each imputed dataset separately. The third step is to mix all the draws together, thereby obtaining (at least approximately) draws from the posterior distribution that take into account the uncertainty associated with the missing data [22]. Summaries (e.g., median, 2.5th, and 97.5th percentiles) of the mix of draws from across the imputed datasets are then obtained for one's parameter estimates. (This third step is perhaps surprising to those familiar with MI inference: Rubin's combining rules are not needed.) Gelman et al. (2004)'s three‐step procedure appears straightforward and might work particularly well in scenarios when the “imputer” and “analyst” are different people [14]. However, when SVL (or “on‐the‐fly”) imputation is used for imputing a derived *outcome* variable in the first step, things can be complicated. This becomes apparent when considering the assumptions required.

Using SVL or “on‐the‐fly” imputation in the first step requires one to specify distributions for each of the missing source variables. Then, specifying the posterior distribution in the second step requires one to define a distribution, along with priors for the parameters that characterize the distribution, for the derived outcome variable. Clearly, the assumed distributions of the source variables (specified in step 1) necessarily determine the distribution of the derived outcome variable (which must be specified in step 2). Without careful consideration, one could inadvertently define incompatible distributions, since the two steps are done separately and the software implementing one step (e.g., MICE from [23]) does not know of the assumptions required by the other step (e.g., rstan from [24]). Moreover, it will no doubt be difficult to determine whether or not the assumptions made by the Bayesian model specified in the second step (e.g., regarding priors) are consistent with the assumptions made when imputing missing values in the first step.

Ideally, one could fit a single fully integrated Bayesian model to simultaneously impute missing values and sample from the posterior. The idea of avoiding potential problems with MI by adopting a fully integrated Bayesian approach is not new, and it is a rather common approach [14, 25, 26, 27, 28]. However, it is not at all obvious how to proceed in this way when there is missingness in source variables that combine to define a derived outcome variable. In this article, we propose a useful approach. In Section 2, we begin with a simple illustrative example. In Section 3, we outline our proposed approach. In Section 4, we consider two applied examples:

1. comparing the BMI of Dutch boys from inside the city to those from outside the city, and
2. estimating the risk of microcephaly in newborns exposed to the ZIKA virus with an artificial dataset. Finally, we conclude in Section 5.

## 2 A Simple Illustrative Example

We continue with the simple example discussed in the introduction where $Y=Z_{1}+Z_{2}$. Suppose we are interested in two groups, group A and group B (e.g., placebo and treatment), and we are interested in estimating the difference between the mean of Y in group A, and the mean of Y in group B. Table 2 lists the data for eight individuals to illustrate, with “NA” indicating missing. The group membership is observed for most individuals but missing for some, and Y is necessarily missing whenever either $Z_{1}$ or $Z_{2}$ are missing.

**TABLE 2:** Eight observations from the hypothetical dataset considered in the example.

i	X (Group)	Y	$Z_{1}$	$Z_{2}$
1	A	NA	NA	1.71
2	A	1.59	−0.09	1.68
3	A	NA	1.23	NA
4	B	2.40	2.20	0.20
5	B	4.49	2.35	2.14
6	B	3.52	0.49	3.03
7	NA	2.00	−0.03	2.03
8	B	NA	1.01	NA

If the data were entirely observed, the easiest way to proceed might be to fit a simple univariate Normal model for Y where, for i=1,…,n:

(1) $$\begin{array}{l} Y_{i}∼\text{Normal}(\alpha +\beta X_{i},\sigma_{Y}^{2}),\; \end{array}$$

where $X_{i}=0$ if observation i is in group A, and $X_{i}=1$ if observation i is in group B, with priors specified for α, β and $\sigma_{Y}$. The estimand (the target quantity that is to be estimated) then coincides with parameter β. However, given that a proportion of Y, X, $Z_{1}$ and $Z_{2}$ values are missing, how should we proceed?

As discussed in the introduction, one could consider the complete‐case analysis, where all rows of data with any missing values are discarded (individuals i=1, i=3, i=7, and i=8 listed in Table 2). The advantage of this strategy is that no imputation step is required, and the entire procedure is to simply fit the univariate Normal model. The disadvantage is that this strategy almost certainly involves a loss of efficiency and has the potential for bias.

Alternatively, one could apply Gelman's three‐step procedure with one of the three MICE approaches (either the DVL, SVL, or JAV imputation methods) for imputing the missing Y and X values, followed by the univariate Normal model for inference. The advantage of this strategy is that, depending on the specific MICE approach used, one could conceivably obtain unbiased estimates of β in an efficient manner. The disadvantage is that this strategy involves two separate steps (an imputation step and an inference step), which may not be consistent with one another.

There is another possible strategy worth considering. Because the mathematics in this scenario are rather simple, one could fit a Bayesian model for the source variables, $Z_{1}$ and $Z_{2}$, and derive the “implied” samples of β. For instance, we could fit a Bernoulli model for X and a bivariate Normal model for $Z_{1}$ and $Z_{2}$, such that, for i in 1,…,n:

(2) $$\begin{array}{ll} X_{i} & ∼\text{Bernoulli}(\zeta ), \end{array}$$

(3) $$\begin{array}{ll} \left(Z_{1,i},Z_{2,i}\right) & ∼\text{Normal}(\mu_{i},\sum ), \end{array}$$

where

(4) $$\begin{array}{ll} & \mu_{i}=\left\{\begin{array}{ll} (\mu_{Z1,A},\mu_{Z2,A})\; & \text{if}\;i\;\text{is in group}\;A\\ (\mu_{Z1,B},\mu_{Z2,B})\; & \text{if}\;i\;\text{is in group}\;B \end{array}\right.\\ & \;\text{and}\;\;\sum =\left(\begin{array}{cc} \sigma_{Z1}^{2} & \rho \sigma_{Z1}\sigma_{Z2}\\ \rho \sigma_{Z1}\sigma_{Z2} & \sigma_{Z2}^{2} \end{array}\right),\\ & \;\text{and, for each MCMC iteration, compute}\\ & \beta =(\mu_{Z1,A}+\mu_{Z2,A})-(\mu_{Z1,B}+\mu_{Z2,B}). \end{array}$$

Priors would need to be specified for each of the eight parameters: ζ, $\mu_{Z1,A}$, $\mu_{Z2,A}$, $\mu_{Z1,B}$, $\mu_{Z2,B}$ $\sigma_{Z1}$, $\sigma_{Z2}$ and ρ.

This strategy might be ideal in the sense that it does not require a separate imputation step (the entire procedure is to simply fit the above model) and does not disregard a large number of observations. However, this approach is only possible because in this simple illustrative example, the mathematics are extremely straightforward (i.e., Equation (4) is known). For other derived variables, this might not be the case. For instance, if instead of $Y=Z_{1}+Z_{2}$, we had $Y=Z_{1}/({Z_{2}}^{2})$, then computing β from a combination of the eight model parameters (ζ, $\mu_{Z1,A}$, $\mu_{Z2,A}$, $\mu_{Z1,B}$, $\mu_{Z2,B}$ $\sigma_{Z1}$, $\sigma_{Z2}$ and ρ) might be impossible without a degree of mathematical wizardry.

One might question why we cannot impute in the missing values and simultaneously estimate β directly within the Bayesian model (with the univariate Normal model). Unfortunately, this will not work since when the missing values are derived outcomes, only a single generative model can be specified. In other words, a missing data model for the source variables and a separate outcome model for the derived variable cannot be expressed as a single generative model for the observable data (e.g., JAGS will return a compilation error: “attempt to redefine node”).

This brings us to our proposed method, details of which are provided in the next section. Briefly, the approach involves using Monte Carlo integration to derive samples of the derived outcome variable. In our example, this would involve fitting the bivariate Normal model and sampling β (at least approximately) without requiring any knowledge about the mathematical derivation of β.

## 3 Description of the Approach

Having established the motivation behind our proposed method, we now outline the details. Suppose the data consists of n observations, and let W be the set of p+q variables at hand. Let $Y=f(W)=f(W_{source})$ be the outcome variable, where $W_{source}$ are the p source variables that combine in some deterministic way to equal the outcome variable. (To be clear, there are p source variables which make up the outcome Y). Let $W_{expl}$ consist of the remaining q explanatory variables. In practice, note that certain variables could be both source variables and explanatory, but we set this aside to avoid more complicated notation. Here, $\dim(W_{source})=(n,p)$ and $\dim(W_{expl})=(n,q)$.

Say W partitions as variables $W_{1}$ which are prone to missingness, and variables $W_{2}$ which are not: $W=\{W_{1},W_{2}\}$. Specifically, $W_{1}$ consists of certain observations that are missing, $W_{1}^{\left\{mis\right\}}$, and certain observations that are observed, $W_{1}^{\left\{obs\right\}}$, such that $W_{1}=\{W_{1}^{\left\{mis\right\}},W_{1}^{\left\{obs\right\}}\}$. We are interested in the situation in which there is overlap between $W_{1}$ and $W_{source}$, that is, situations when certain source variables are subject to missingness. Let $W_{1,source}=W_{1}\cap W_{source}$, $W_{1,expl}=W_{1}\cap W_{expl}$, $W_{2,source}=W_{2}\cap W_{source}$, and $W_{2,expl}=W_{2}\cap W_{expl}$.

In the simple illustrative example we have $W=\{Z_{1},Z_{2},X\}$ (where X is the binary group indicator variable such that, for the i‐th observation, $X_{i}=0$ if i‐th observation is in Group A and $X_{i}=1$ if the i‐th observation is in Group B). Furthermore, we have $W_{1}=\{Z_{1},Z_{2},X\}$, $W_{2}=\{\emptyset \}$, p=2, $W_{source}=\{Z_{1},Z_{2}\}$, q=1, $W_{expl}=\{X\}$, and $Y=f(W)=Z_{1}+Z_{2}$.

Typically, the objective of a Bayesian analysis consists of the estimation of an estimand [29]. The estimand of interest, θ, is often defined nonparametrically as a function of certain conditional expectations: $\theta =g(E_{1}^{*}\{E(Y|W_{expl})\},\ldots ,E_{D}^{*}\{E(Y|W_{expl})\})$, where $E_{d}^{*}$ corresponds to the counterfactual expectation with respect to the d‐th population of interest for d in 1,…,D.

In certain situations, the D population(s) of interest might themselves be unknown and require estimation. For example, suppose the estimand of interest is the mean difference in Y between those receiving treatment (X=1) and those not receiving treatment (X=0) for individuals who are of “average age,” where average age, $\mu_{A}$, is unknown. Then, we have D=2 populations of interest: (1) treated individuals of average age, and (2) untreated individuals of average age. The estimand is defined as: $\theta =E(Y|X=1,A=\mu_{A})-E(Y|X=0,A=\mu_{A})$. The parameter $\mu_{A}$ is required to define the two populations of interest and must be estimated from the data.

In the simple illustrative example, the estimand of interest is the difference in the mean of Y between individuals in the two groups (i.e., the difference between the conditional expectation of Y given the D=2 populations of interest defined by X=0 and X=1): θ=E(Y|X=1)−E(Y|X=0). Because the mathematics are conveniently simple, one can also define this estimand analytically as a function of the model parameters: θ=β, but also as: $\theta =h(\phi_{source})=(\mu_{Z1,A}+\mu_{Z2,A})-(\mu_{Z1,B}+\mu_{Z2,B})$, where $\phi_{source}=\{\zeta ,\mu_{Z1,A}$, $\mu_{Z2,A}$, $\mu_{Z1,B}$, $\mu_{Z2,B}$ $\sigma_{Z1}$, $\sigma_{Z2}$, ρ}. The standard Bayesian approach would then be to obtain a Monte Carlo sample of size M (e.g., via MCMC) from the $\left(\phi_{source},W_{1}^{\left\{mis\right\}}|W_{1}^{\left\{obs\right\}},W_{2}\right)$ posterior distribution and then, since h(·) is known, obtain M posterior samples of θ by applying the h(·) function to each of the M posterior samples of $\phi_{source}$.

In situations when the mathematics are not so simple, Bayesian g‐computation [30, 31] can be used for a form of “model‐based standardization” [32, 33] in order to obtain posterior samples of θ. Bayesian g‐computation has been suggested previously for resolving problems with missing data, but not in the context of missingness with derived outcome variables [34].

The Bayesian g‐computation approach proceeds according to the following steps.

1. Draw M samples of $\left(\phi_{source},W_{1,expl}^{\left\{mis\right\}}\right)$ from the $\left(\phi_{source},W_{1,expl}^{\left\{mis\right\}}|W_{1}^{\left\{obs\right\}},W_{2}\right)$ distribution: ${\left(\phi_{source},W_{1,expl}^{\left\{mis\right\}}\right)}^{\left[m\right]}$, for m in 1,…,M.
2. For m in 1,…,M:
  a. For d in 1,…,D:
    i. If the d‐th population of interest is defined in terms of unknown parameters, $\gamma^{d}$, these must be estimated from the data. Obtain a draw from $\gamma^{d}|{\left(\phi_{source},W_{1,expl}^{\left\{mis\right\}}\right)}^{\left[m\right]}$: $\gamma^{d[m]}$.
    ii. Draw S (where S is a large number) iid samples from the $\left(W_{source}|\gamma^{d[m]}\right)$: $W_{source}^{*d[m,s]}$, for s in 1,…,S. Note: $\dim(W_{source}^{*d[m,s]})=(1,p)$.
    iii. For s in 1,…,S: Apply the deterministic function f(·) to $W_{source}^{*d[m,s]}$ to obtain a sample from the $\left(Y|\gamma^{d[m]}\right)$ distribution: $Y^{*d[m,s]}=f(W_{source}^{*d[m,s]})$. Note: $\dim(Y^{*d[m,s]})=(1,1)$.
    iv. Calculate (approximately) the sample mean: $E_{d}^{*}{\left\{E(Y|W_{expl})\right\}}^{\left[m\right]}\approx \frac{1}{S}\sum_{s=1}^{S}Y^{*d[m,s]}$.
  b. Obtain $\theta^{\left[m\right]}$ by combining $E_{1}^{*}\{E(Y|W_{expl})\},\ldots ,E_{D}^{*}\{E(Y|W_{expl})\}$ according to the definition of the estimand of interest.

Having obtained M posterior samples of θ following the above steps, one can calculate the posterior median/mean and credible interval for θ (or perhaps a Bayes factor comparing two sub‐models of interest [35]), as is standard practice in a Bayesian analysis. Note that both M and S should be sufficiently large that *both* sources of Monte Carlo error are negligible. To be clear, the posterior distribution is estimated simultaneously for both the unknown parameters and for the missing data, so all sources of uncertainty (including the uncertainty in the imputed values) properly propagate into the estimate of θ.

In the simple illustrative example, the D=2 populations of interest were defined by X=0 and X=1. Therefore the m‐th posterior sample of θ would be obtained in Step 2(b) as:

(5) $$\theta^{\left[m\right]}=\frac{1}{S}\overset{S}{\underset{s=1}{\sum }}Y^{*2[m,s]}-\frac{1}{S}\overset{S}{\underset{s=1}{\sum }}Y^{*1[m,s]},$$

where $Y^{*1[m,s]}$ is a sample from the distribution of $\left(Y|\phi_{source}^{\left[m\right]},X=0\right)$ and $Y^{*2[m,s]}$ is a sample from the distribution of $\left(Y|\phi_{source}^{\left[m\right]},X=1\right)$. In general, note that there is no requirement to explicitly define two populations of interest (i.e., groups A and B). Indeed, there are no restrictions imposed on how the estimand, θ, must be defined.

## 4 Applied Examples

We demonstrate our proposed approach with two applied examples. The Dutch Boys example was chosen as an accessible textbook example that is often used to demonstrate how various imputation methods can be applied (e.g., [36, 37]). The second example was chosen as it originally motivated our proposed method. Our proposed approach can be applied with various MCMC software. To demonstrate, we used JAGS [38] for the first example and Stan [24] for the second. All code is available in the Supporting Information.

### 4.1 Multiple Linear Regression of the Body Mass Index of Dutch Boys

#### 4.1.1 Background

The “Growth of Dutch boys” dataset is available as an example dataset within the “mice” package for R [23] and is often used to demonstrate how various imputation methods can be applied. Here we use this dataset to illustrate, in a very accessible way, how the approach outlined in Section 3 could be applied for determining the difference in logBMI between boys from the “city” and boys from outside of the “city,” when controlling for age.

The outcome of interest, Y=log(BMI), is a derived continuous variable, based on two source variables:

1. height ($Z_{1}$, measured in centimeters),
2. weight ($Z_{2}$, measured in kilograms). This is derived as follows:
(6) $$Y=\text{logBMI}=f(Z_{1},Z_{2})=\log\left(\frac{Z_{2}}{{\left(Z_{1}/100\right)}^{2}}\right).$$
  The regional status variable (X, equal to 1, if the boy is from the “city” and equal to 0 otherwise) is of particular interest. We also consider the variable age (A, measured in years) as highly predictive of $Z_{1}$, $Z_{2}$, and Y.

#### 4.1.2 The Data

**FIGURE 1:**
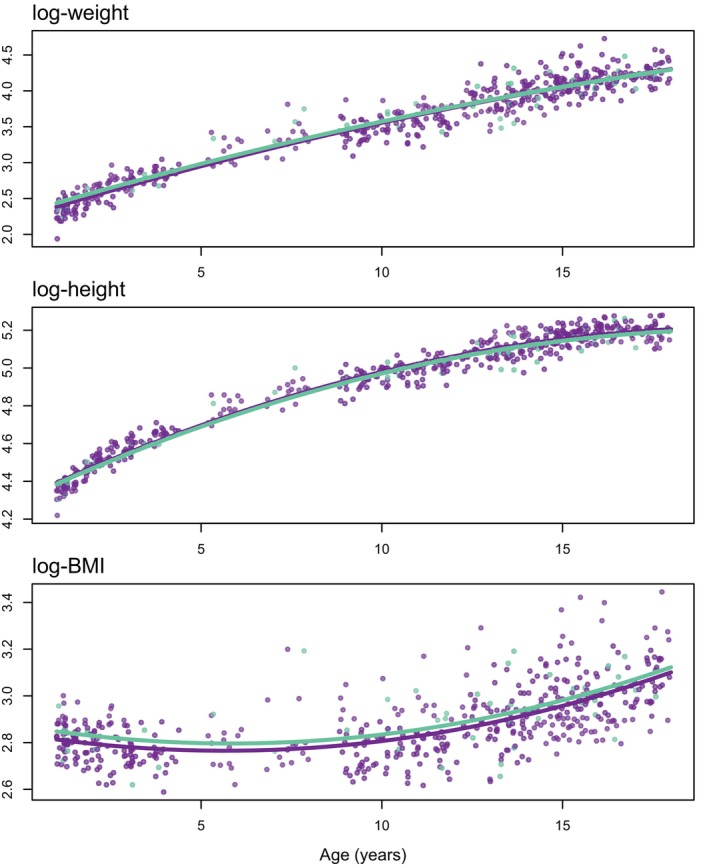
The complete‐case data for the Dutch boys analysis (n=518) with violet dots corresponding to boys from outside the city (X=0) and green dots corresponding to boys from within the city (X=1). Lines drawn from least‐squares estimates.

The data consists of measurements on a representative sample of n=537 boys between the ages of 1 and 18 years (inclusive), of which approximately 9% are from the “city” (we restricted the original “Growth of Dutch boys” dataset (n=748) to the 1‐18 age range to allow for reasonable modeling of how log(BMI) changes with age). Figure 1 plots the data among the complete cases. All variables, except age (A), are subject to a small amount of missingness. The regional status variable, “city” (X), is missing for 1 subject; height is missing for 18 subjects; and weight is missing for 2 subjects. Overall, 19 out of the 537 observations (3.5%) have at least one missing value. Following the notation defined in Section 3, we have that $W_{1}=\{Z_{1},Z_{2},X\}$, $W_{2}=\{A\}$, p=2, $W_{source}=\{Z_{1},Z_{2}\}$, q=2, and $W_{expl}=\{X,A\}$. Note that the missingness of height and weight may or may not depend on age and/or regional status.

The primary objective of the study is to estimate the difference in mean logBMI between boys from inside the “city” and boys from outside of the “city,” when controlling for age. A secondary objective is to make probabilistic predictions of logBMI for boys given their age and regional status.

#### 4.1.3 The Estimand

Based on the primary objective, the estimand of interest is the difference in mean logBMI between boys from the “city” and boys from outside of the “city,” when controlling for age, and this can be defined as a function of the conditional expectation of Y given D=2 populations of interest:

(7) $$\begin{array}{ll} \theta & =\int_{a}E(Y|X=1,A=a)f_{A}(a)da-\int_{a}E(Y|X=0,A=a)f_{A}(a)da, \end{array}$$

where $f_{A}(a)$ corresponds to the distribution of ages among boys in the Netherlands. Since the data supposedly consist of a representative sample of Dutch boys, we assume that $f_{A}(a)$ also coincides with the sample distribution of ages.

#### 4.1.4 Two Models

The univariate normal model involves considering the logBMI of each subject as the outcome of a simple univariate Normal model:

(8) $$(Y|X=x,A=a)∼\text{Normal}(\beta_{0}+\beta_{1}x+\beta_{2}a+\beta_{3}xa+\beta_{4}a^{2},\sigma^{2}),$$

and:

X∼Bernoulli(π).

This model assumes a quadratic relationship between BMI and age, a reasonable approximation based on published growth curves [39] and allows for the possibility that age is an effect modifier with respect to the effect of “city.” For the univariate normal model, we set standard Normal priors, Normal(0,1), for the regression coefficient parameters $\beta_{0}$, $\beta_{1}$, $\beta_{2}$, $\beta_{3}$ and $\beta_{4}$, an exponential prior, Exponential(1), for the standard deviation parameter, σ, and a uniform prior, Uniform[0,1], for π.

The bivariate normal model considers height and weight as two correlated outcomes such that:

(9) $$\begin{array}{ll} \left(\log(Z_{1}),\log(Z_{2})|X=x,A=a\right) & ∼\text{Normal}(\mu_{i},\sum ), \end{array}$$

where

(10) $$\begin{array}{ll} \mu_{i} & =\left(\begin{array}{c} \alpha_{0}+\alpha_{1}x+\alpha_{2}a+\alpha_{3}xa+\alpha_{4}a^{2}\\ \gamma_{0}+\gamma_{1}x+\gamma_{2}a+\gamma_{3}xa+\gamma_{4}a^{2} \end{array}\right),\;\;\\ \sum & =\left(\begin{array}{cc} \tau_{Z1}^{2} & \rho \tau_{Z1}\tau_{Z2}\\ \rho \tau_{Z1}\tau_{Z2} & \tau_{Z2}^{2} \end{array}\right), \end{array}$$

and:

(11) X∼Bernoulli(π).

We set standard Normal priors, Normal(0,1), for all the regression coefficient parameters $\alpha_{0}$, $\alpha_{1}$, $\alpha_{2}$, $\alpha_{3}$, $\alpha_{4}$, $\gamma_{0}$, $\gamma_{1}$, $\gamma_{2}$, $\gamma_{3}$, and $\gamma_{4}$; exponential priors, Exponential(1), for the standard deviation parameters, $\tau_{Z1}$ and $\tau_{Z2}$, a uniform prior, Uniform[−1,1], for the correlation parameter, ρ, and another uniform prior, Uniform[0,1], for π.

We can define the estimand of interest analytically as a function of the parameters of the univariate normal model:

(12) $$\begin{array}{ll} \theta =h_{1}(\eta ) & =\int_{a}(\beta_{1}+\beta_{3}a)f_{A}(a)da \end{array}$$

(13) $$\begin{array}{ll} & \;=\beta_{1}+\beta_{3}E(A), \end{array}$$

where $\eta =\{\beta_{0},\beta_{1},\beta_{2},\beta_{3},\beta_{4},\sigma \}$. Since the data are assumed to be a representative sample from the population of interest, and since we are choosing not to model the distribution of age (not required because there are no missing values for age), we can substitute E(A) for the sample mean, Ā.

Coincidentally, we can define the estimand as a function of the parameters of the bivariate normal model (see details in the Appendix):

(14) $$\begin{array}{ll} \theta =h_{2}(\phi ) & =\int_{a}(\gamma_{1}+\gamma_{3}a-2\alpha_{1}-2\alpha_{3}a)f_{A}(a)da\\ & =\gamma_{1}-2\alpha_{1}+(\gamma_{3}-2\alpha_{3})E(A), \end{array}$$

where $\phi =\{\alpha_{0},\alpha_{1},\alpha_{2},\alpha_{3},\alpha_{4},\gamma_{0},\gamma_{1},\gamma_{2},\gamma_{3},\gamma_{4},\tau_{Z1},\tau_{Z2},\rho \}$, and we once again can substitute E(A)=Ā.

#### 4.1.5 Methods and Results

We conducted the analysis with four different approaches:

1. Fitting the univariate normal model to the complete‐case data and applying the $h_{1}(\cdot )$ function to obtain posterior draws of θ (with M=2000).
2. Imputation of missing values with “on‐the‐fly” MI (including a quadratic term for age and age‐city interaction terms in the imputation model) followed by fitting the univariate normal model (Equation (8)) with Gelman's three‐step approach, and applying the $h_{1}(\cdot )$ function to obtain posterior draws of θ (with M=2000, and K=50 imputed datasets).
3. Fitting the bivariate normal model and applying $h_{2}(\cdot )$ function to obtain posterior draws of θ (with M=2000).
4. Fitting the bivariate normal model and applying the proposed method detailed in Section 3 (with M=2000, and S=2000).

**FIGURE 2:**
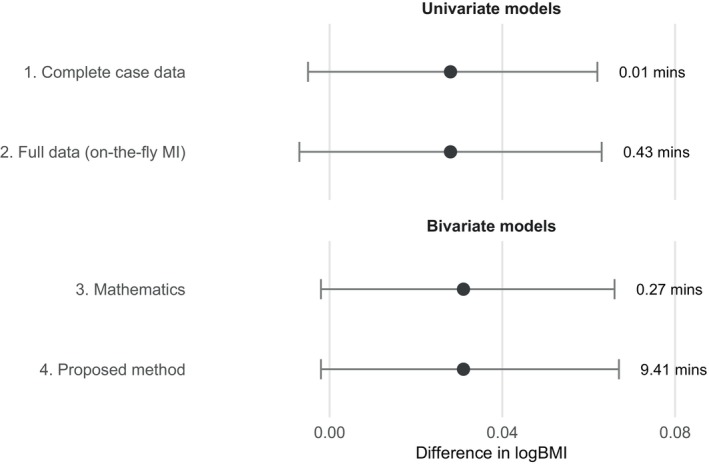
Results for the analyses of the Dutch boys dataset. Estimates list the posterior median estimates (i.e., the median of the posterior estimates of the mean difference in logBMI) and equal‐tailed posterior 95% CrIs. Labels indicate the computational time required for each method in minutes.

Both the univariate model and bivariate model were fit using JAGS with 1000 burn‐ins [38]. R and JAGS code is available in the Appendix. Results are listed in Figure 2. The estimate from the complete‐case analysis is similar to the estimate obtained when using the univariate normal model with Gelman's three‐step approach with “on‐the‐fly” MI. This is not surprising given the small amount of missingness (only 3.5% of observations have a missing value). Estimates are also similar when using the bivariate model. The estimate obtained with fitting the bivariate normal model and applying $h_{2}(\cdot )$ function is slightly more precise than the estimate obtained with fitting the bivariate normal model and applying the proposed method (compare [−0.002, 0.066] vs. [−0.002, 0.067]), suggesting that there is a minuscule cost to the numerical approximation (even with the large S=2000). With respect to computational time, the proposed method is substantially more time‐consuming than the other methods. However, note that the g‐computation could be parallelized, and the small cost (in terms of the increased width of the credible interval) could be reduced/removed by making S larger.

### 4.2 Estimating the Risk of Microcephaly

#### 4.2.1 Background

ZIKA virus infection (ZIKV) during pregnancy is known to be associated with an increased risk of fetal congenital malformations, including microcephaly, a birth condition in which a baby's head is smaller than expected when compared to babies of the same sex and age. Formally, microcephaly is defined as a measure of head circumference at birth of more than 2 standard deviations below average for sex and gestational age [40]. (Average values for head circumference at birth are determined by referencing the standards calculated by the intergrowth‐21st research network [41]). Therefore, in a healthy population, we would anticipate 2.28% of newborns being categorized as having microcephaly (assuming head circumference in the general population is Gaussian).

In order to determine the risk of microcephaly associated with ZIKV in mothers, observational data have been collected by numerous studies from infected pregnant women [42]. These data typically suffer from substantial missingness.

The outcome of interest, microcephaly (Y), is a derived binary variable, based on three source variables: (1) sex ($Z_{1}$ = 0 for “male” and $Z_{1}$ = 1 for “female”), (2) gestational age ($Z_{2}$, measured in weeks), and (3) head circumference ($Z_{3}$, measured in centimeters). Each of these three source variables is subject to missingness. The estimand of interest is the probability of microcephaly (θ=E(Y)) given the assumed (D=1) population of interest from which the data are sampled.

#### 4.2.2 The Data and Models

**FIGURE 3:**
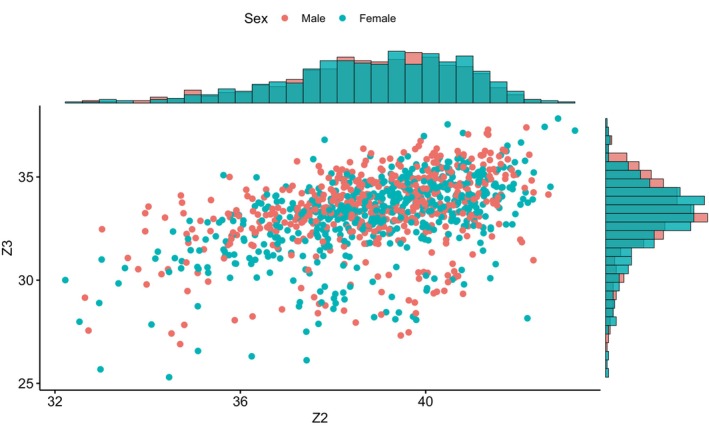
The complete‐case data (n = 1019) with values (1) sex ($Z_{1}$ = 0 for “male” and $Z_{1}$ = 1 for “female”), (2) gestational age ($Z_{2}$, measured in weeks), and (3) head circumference ($Z_{3}$, measured in centimeters).

We consider a simple synthetic dataset based on what is typically observed in observational studies. The purpose of this analysis is to demonstrate all of the steps required when following each of the different approaches for dealing with missingness based on the problem that initially motivated our research. As such, we stress that the specific details of how the data were created are less important and acknowledge that a different dataset would no doubt lead to different results. In brief, the data are simulated with a “true” risk of microcephaly of θ = 11.69%. The data represent sex ($Z_{1}$), gestational age ($Z_{2}$), and head circumference ($Z_{3}$) measurements on n=1800 newborns with at least one of $Z_{1},Z_{2},Z_{3}$ observed; Figure 3 shows head circumference and gestational age among those with both observed. Missing sex values are attributed completely at random, missing gestational age values are more likely for those with very small head circumference values, and missing head circumference values are more likely for those with higher gestational age. This could be due to a higher likelihood of measuring the head circumferences of premature babies, who are often considered higher risk.

**FIGURE 4:**
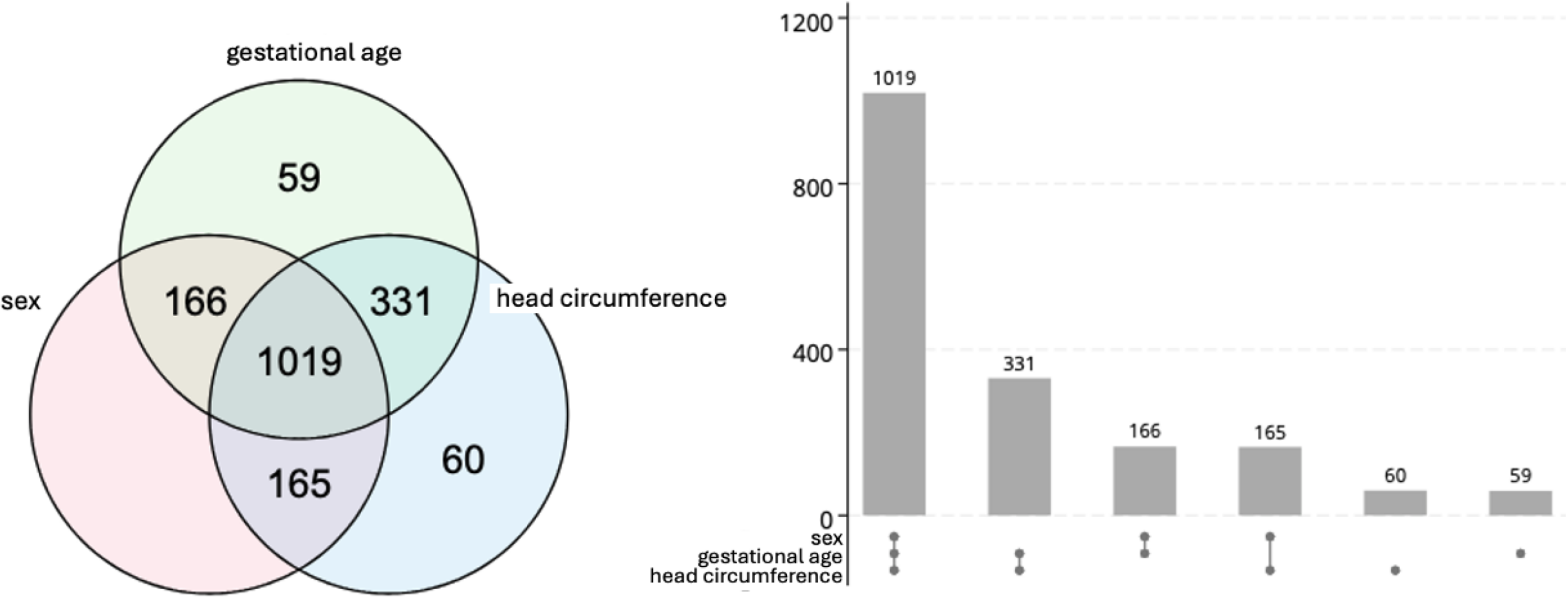
Venn diagram and UpSet plot indicating the number of observations in the dataset with observed data. There are n = 1019 individuals for whom there is complete data, and n = 781 individuals who have at least one value missing.

There are 781 individuals who have at least one value missing; see the Venn diagram in Figure 4. Microcephaly status, Y, for a newborn can be calculated by first deriving their “z‐score” based on their head circumference, gestational age, and sex. (The “igb_hcircm2zscore” function in the growth standards R package can do this easily using the intergrowth standards). If a newborn's z‐score is below −2, they are classified as having microcephaly. As such, while the f(·) function is known and can be easily applied to the source variables to obtain the binary derived variable, it is not easily written out.

We consider two models:

1. a simple “Bernoulli model,” and
2. a “Bernoulli skew‐Normal mixture‐Normal model” (BsNmN model). Both models were fit using Stan with M=5000 draws (6000 iterations and 1000 burn‐ins) [24]; Stan code is available in the Appendix.

The Bernoulli model involves considering the binary microcephaly status of each individual as the outcome of a simple Bernoulli model where θ is the risk of microcephaly:

(15) Y∼Bernoulli(θ).

**FIGURE 5:**
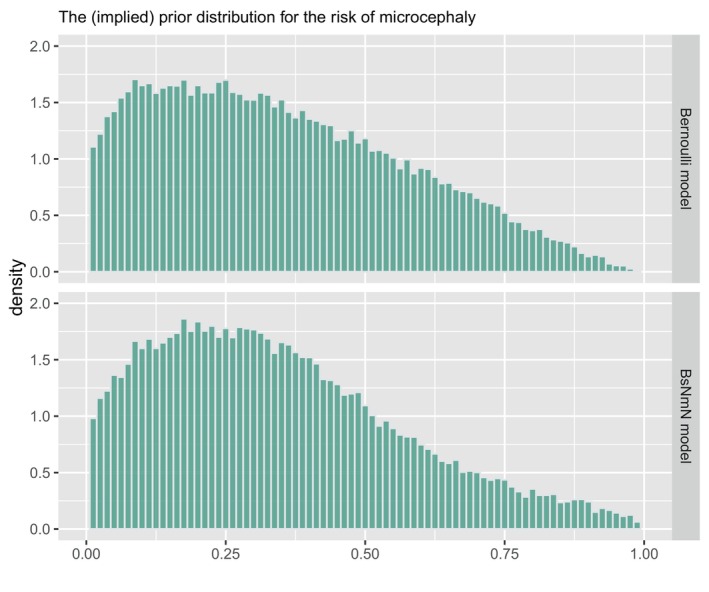
Lower panel shows histogram of a sample from the “implied prior” on E(Y) used in the BsNmN model for the microcephaly example analysis. Upper panel shows histogram of a sample from the Beta(1.26, 2.32) prior placed on θ used in the Bernoulli model. The numbers 1.26 and 2.32 were specifically chosen so that these two priors would be similar.

We set a Beta(1.26,2.32) prior for θ (see top panel of Figure 5), corresponding to an a priori belief that Pr(θ<31.5%)=50%. (This prior was selected so that the two models have (implied) priors matching as closely as possible; see Figure 5).

We fit the Bernoulli model to the complete‐case data (n=1019) and obtained an estimate of: $\hat{\theta }=9.18\%$, 95% CrI = [7.60%, 10.92%] (posterior median and equal‐tailed 95% credible interval). We also fit the Bernoulli model to the entire dataset (n = 1800) and followed Gelman's three‐step procedure with SVL imputation. Specifically, we used MICE specifying the “logreg” method for $Z_{1}$ and the “normal” method for $Z_{2}$ and $Z_{3}$. For each of K=50 imputed datasets, we fit the Bernoulli model using MCMC with M = 5000 draws, and after combining all 250 000 draws together, we obtained a posterior median estimate of: $\hat{\theta }=12.70\%$, with equal‐tailed 95% CrI = [11.00%, 14.51%].

The BsNmN model involves a series of conditional distributions for each of the three source variables. We assume that an infant's sex ($Z_{1}$) is unlikely to impact their gestational age at birth ($Z_{2}$) (at least by any appreciable amount; see [43]). Therefore, for sex, we define:

(16) $$Z_{1}∼\text{Bernoulli}(0.5).$$

The distribution of gestational age ($Z_{2}$) is known to be skewed with a long left tail (due to preterm delivery), almost complete truncation on the right tail at 45 weeks (due to medically induced labor at around or before 45 weeks) and almost complete truncation on the left tail at around 24 weeks (due to viability). Many complex distributions have been suggested for modeling gestational age (e.g., Rathjens et al. (2024) [44] recommend the three‐parameter Dagum distribution). Following Sauzet et al. (2015) [45], we choose to define a skew‐Normal distribution centered at 39 weeks:

(17) $$(Z_{2}-39)∼\text{skew‐Normal}\left(\mu -\sqrt{\frac{2}{\pi }}\frac{\sigma \omega }{\sqrt{1+\omega^{2}}},\sigma ,\omega \right),$$

where μ is the mean of the distribution (and mean gestational age known to be approximately 39 weeks, according to [45]), σ is the scale, and ω is the slant.

**FIGURE A2:**
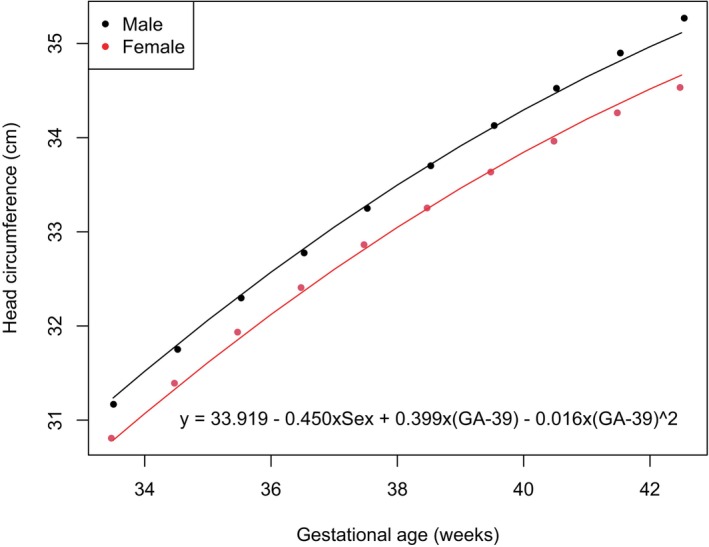
The quadratic regression model fit to digitized data from Villar et al. (2014) [41]'s Figure 2C for the microcephaly example analysis.

Finally, head circumference ($Z_{3}$) is known to be approximately Gaussian and mean head circumference increases in an approximately quadratic way with gestational age (see Figure 2C in [41], and Figure A2 in the Appendix). In a ZIKV‐infected population, infection is thought to cause a certain proportion of infants (i.e., “affected” individuals) to have smaller heads. As in Kalmin et al. (2019) [46], we define a Normal mixture distribution for head circumference ($Z_{3}$):

(18) $$\begin{array}{ll} Z_{3}|Z_{1} & =z_{1},\;Z_{2}=z_{2}\\ & ∼q\text{Normal}(\beta_{0}+\beta_{1}z_{1}+\beta_{2}(z_{2}-39)+\beta_{3}{\left(z_{2}-39\right)}^{2},\zeta_{1})\\ & \;+(1-q)\text{Normal}(\kappa +\beta_{1}z_{1}+\beta_{2}(z_{2}-39)+\beta_{3}{\left(z_{2}-39\right)}^{2},\zeta_{2}), \end{array}$$

where q is the proportion of “non‐affected” individuals and (1−q) is the proportion of “affected” individuals. The $\beta_{0}$, $\beta_{1}$, $\beta_{2}$ and $\zeta_{1}$ parameters relate the distribution of head circumference to sex and gestational age for the “non‐affected” population; and the κ and $\zeta_{2}$ parameters correspond to the impact of ZIKV for the “affected” individuals in terms of the difference in mean head circumference and the scale.

The parameters in the BsNmN model are $\psi =(\mu ,\sigma ,\omega ,\beta_{0},\beta_{1},\beta_{2},\beta_{3},\zeta_{1},\zeta_{2},\kappa ,q)$ and each requires a prior. Based on the information available in [41] and [45], we can be reasonably certain of how gestational age and head circumference are distributed in the nonaffected population and therefore set the following informative priors:

$$\begin{array}{lll} & \mu ∼\text{Normal}(0,0.1),\; & \\ & \beta_{0}∼\text{Normal}(33.912,0.1),\;\; & \beta_{1}∼\text{Normal}(-0.450,0.1),\\ & \beta_{2}∼\text{Normal}(0.399,0.1),\;\text{and}\; & \beta_{3}∼\text{Normal}(-0.016,0.1). \end{array}$$

For the scale and slant parameters, we set the following weakly informative priors:

$$\begin{array}{lll} & \sigma ∼\text{inv‐Gamma}(2,2),\; & \omega ∼\text{Normal}(0,2),\\ & \zeta_{1}∼\text{inv‐Gamma}(2,2),\text{and}\; & \zeta_{2}∼\text{inv‐Gamma}(2,2). \end{array}$$

Finally, a truncated Normal prior for κ was chosen in an effort to help address issues with identifiability that are common when fitting Bayesian mixture models [47], and a uniform prior was chosen for w:

$$\kappa ∼\text{Normal}{\left(-2,2\right)}_{\left[,-1\right]},\;q∼\text{Uniform}(0,1).$$

**FIGURE 6:**
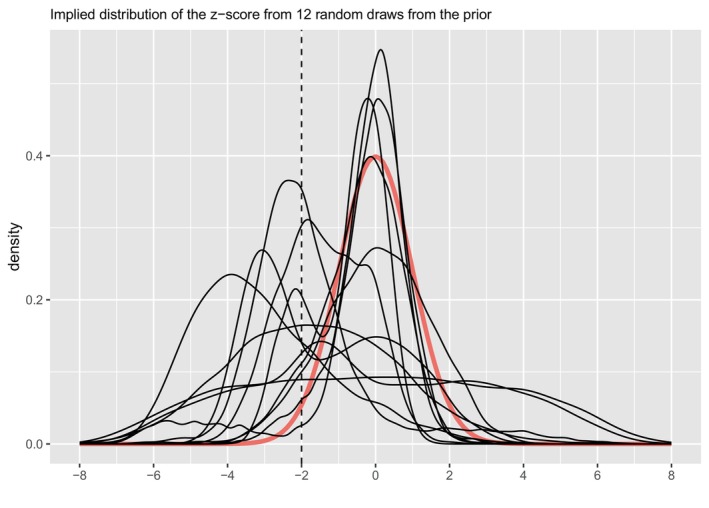
The implied distribution of the z‐score from 12 random draws from the prior of the BsNmN model for the microcephaly example analysis. For reference, the red curve corresponds to the density of the standard normal.

These priors correspond to an a priori belief that Pr(E(Y)<31.5%)=0.5. The bottom panel of Figure 5 plots the implied prior distribution for E(Y) (i.e., for the risk of microcephaly) and Figure 6 plots the implied distribution of the z‐score from 12 random draws of the prior.

**FIGURE 7:**
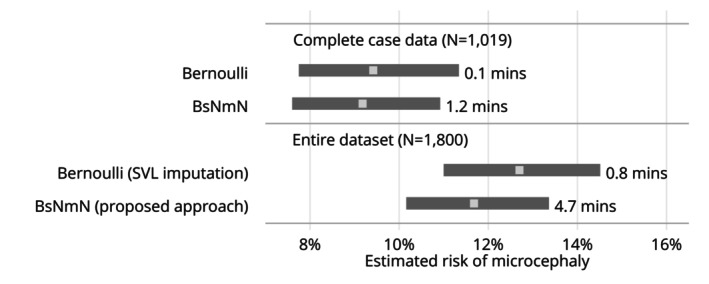
Results from the microcephaly analyses (posterior medians and equal‐tailed 95% credible intervals).

**FIGURE 8:**
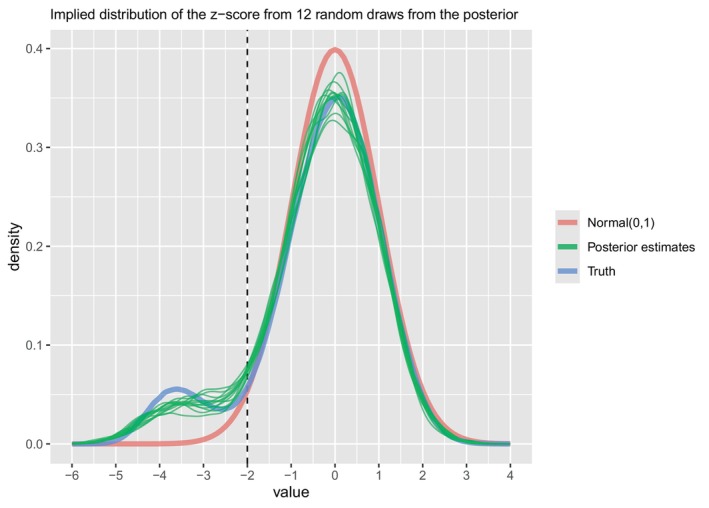
The implied distribution of the z‐score from 12 random draws from the posterior of the BsNmN model. Newborns with z‐scores less than −2 (vertical dashed line) are classified as having microcephaly. For reference, the red curve corresponds to the density of the standard normal, and the blue curve corresponds to the “truth” from which the data were simulated.

**FIGURE A3:**
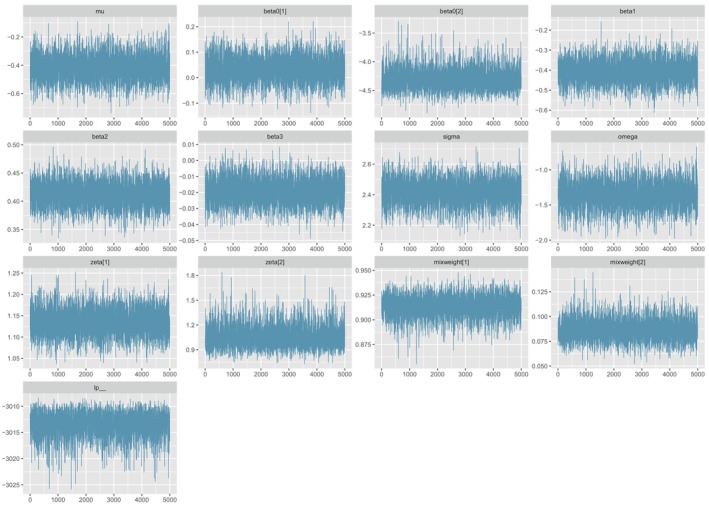
Trace plots from the complete‐case data BsNmN model for the microcephaly example analysis.

**FIGURE A4:**
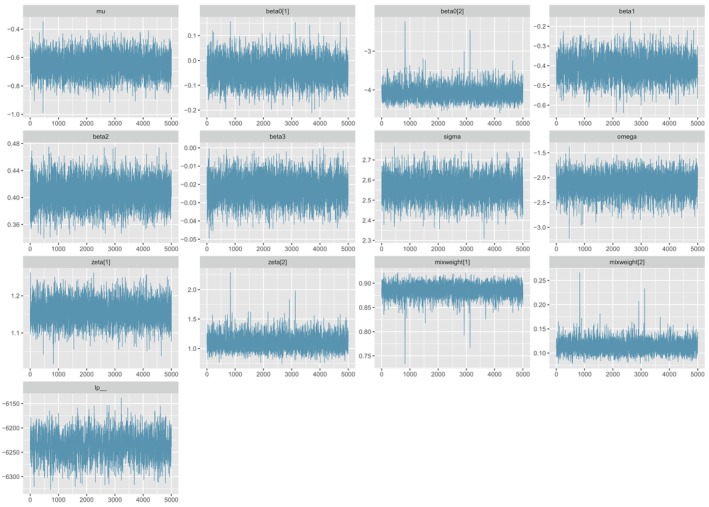
Trace plots from the entire dataset BsNmN model for the microcephaly example analysis.

Figure 7 plots the results of the fitting of the Bernoulli model and the BsNmN model. Fitting the BsNmN model to the complete‐case data (n=1019) we obtain an estimate of: $\hat{\theta }=9.18\%$, 95% CrI = [7.60%, 10.92%]. We also applied the proposed approach to fit the BsNmN model to the entire dataset (n=1800) as detailed in Section 2 with S = 5000 and obtained an estimate of: $\hat{\theta }=11.68\%$, 95% CrI= [10.16%, 13.36%]. Figure 8 plots the implied distribution of the z‐score from the prior and posterior. Figures A3 and A4 in the Appendix show the trace plots of the MCMC. Since we know the truth (θ=11.69%), we can interpret the different results as follows. First, the Bernoulli and BsNmN models fit to the complete‐case data obtain notably biased estimates with the true value outside of the 95% CrIs. When analyses are based on the entire dataset, estimates are better with the BsNmN model, obtaining a point estimate particularly close to the truth. Since this is not a simulation study with repeated simulations and analyses, we are cautious not to over‐interpret how these results compare to one another. However, we can be very certain that with respect to computational time, the Bernoulli model (with SVL imputation) is much faster than the proposed method with the BsNmN model (0.8 min vs. 4.7 min).

To illustrate how uncertainty would appropriately propagate to subsequent downstream analyses, consider using microcephaly status to predict risk of developmental delay at age 2 (DD) [48, 49]. Suppose we have an established model for DD (e.g., Pr(DD=1|Y,X)=expit(α+βY+γX), where X represents covariates, and Y represents microcephaly status). Furthermore, suppose microcephaly status is missing for certain individuals (due to missing values of either sex ($Z_{1}$), gestational age ($Z_{2}$), and/or head circumference ($Z_{3}$)). While one might not have access to the original data used to fit the microcephaly risk model, one can use the output of the microcephaly risk model to “impute” these missing values for the DD model. For example, if an individual has unknown gestational age, but known head circumference ($Z_{3}=z_{3}$) and known sex ($Z_{1}=z_{1}$), we would proceed as follows.

For each posterior draw from the microcephaly risk model (i.e., for m=1,…,M draws of $\phi_{source}$), we have S samples of the source variables, $Z_{1}$, $Z_{2}$, and $Z_{3}$ (from in Step 2(a) (ii)). We proceed by selecting the subset of the S samples of $Z_{2}$ for which the corresponding $Z_{1}=z_{1}$ and the corresponding $Z_{3}=z_{3}\pm \epsilon$ (where ϵ is some small value needed to soften the equality, since head circumference is continuous). Let the number of individuals in this subset be $G_{m}$ and note that S should be sufficiently large to ensure that $G_{m}$ is not too small. We then calculate $Y_{z1,z3}^{*[g,m]}$, for g in $1,\ldots ,G_{m}$: the implied microcephaly status for an individual with this value of $Z_{2}$ and with $Z_{1}=z_{1}$, $Z_{3}=z_{3}$. Finally, averaging over the $G_{m}$ values of $Y_{z1,z3}^{*[g,m]}$ provides us with the implied probability of microcephaly for such an individual: ${\hat{p}}_{z1,z3}^{\left[m\right]}=\mathrm{Pr}(Y=1|Z_{1}=z_{1},Z_{3}=z_{3},\psi =\psi^{\left[m\right]})$. The distribution of these values across the M posterior draws can then be used as input for the DD model to derive an implied posterior distribution for the risk of DD (e.g., $\mathrm{Pr}{\left(DD=1|Z_{1}=z_{1},Z_{3}=z_{3},X\right)}^{\left[m\right]}=(1-{\hat{p}}_{z1,z3}^{\left[m\right]})\text{expit}(\alpha +\gamma X)+{\hat{p}}_{z1,z3}^{\left[m\right]}\text{expit}(\alpha +\beta +\gamma X)$, for m in 1,…,M).

## 5 Discussion

Originally motivated by challenges with estimating the risk of microcephaly, we proposed a fully integrated Bayesian approach that requires one to specify a single model for the missing values and the outcome. In our illustrative analysis, we used the approach to fit a microcephaly risk model and obtained posterior draws of microcephaly risk that fully account for the uncertainty in both the source variables (due to missingness) and the model parameters.

While our approach will ensure consistency, it may require additional complexity. For instance, a researcher might be able to make relatively simple assumptions about the distribution of the derived outcome variable (e.g., be able to assume that microcephaly status is Bernoulli), but have difficulty in making reasonable distributional assumptions for the source variables (e.g., what are reasonable distributions to assume for gestational age and head circumference?). The same considerations apply to defining priors. A researcher might be able to define reasonable priors (based on real prior knowledge) for the parameters that define the distribution of the derived outcome variable (e.g., be able to define a Beta prior for θ such that Pr(θ<31.5%)=50% in the microcephaly example), but have difficulty defining the potentially numerous priors required for modeling the source variables (e.g., define appropriate priors for $\psi =(\mu ,\sigma ,\omega ,\beta_{0},\beta_{1},\beta_{2},\beta_{3},\zeta_{1},\zeta_{2},\kappa ,w)$). One possible pragmatic solution might be to define very uninformative priors for the parameters required for modeling the source variables and include additional “synthetic” priors [50] for the parameters that define the distribution of the derived outcome variable. Further research is required to determine if this is feasible.

The advantages of modeling the source variables directly include the ability to model missingness within a fully integrated Bayesian model following our proposed method. Incorporating informed priors specific to the individual source variables might also be an advantage in certain scenarios. One could also incorporate adjustments for other sources of bias beyond missingness, such as preferential sampling [51] and measurement error. This is an important future goal for the microcephaly model, given the important issues considered by Harville et al. (2020) [40].

Finally, we note that our proposed approach allows one to conduct the analysis of data with missingness within an entirely Bayesian framework. In some instances, one might wish to fit a Bayesian imputation model for the missing values and a frequentist model for the main analysis (i.e., for the outcome model). In such a case, Rubin's rules could be appropriately and easily applied. Alternatively, if both the imputation model and the outcome model of interest must be frequentist, von Hippel & Bartlett's rules may be more appropriate [52]. To the best of our knowledge, if the imputation model is frequentist and the outcome model is Bayesian (an admittedly obscure scenario), there does not appear to be a good approach for obtaining estimates.

## Author Contributions

H. C. conceived the idea, H. C., T. P. M. and P. G. developed the methods and wrote and reviewed the manuscript.

## Funding

This work was supported by the NSERC Discovery Grant (Grant No. RGPIN‐2019‐03957) and the MRC grant (Grant No. MC_UU_0000407).

## Conflicts of Interest

The authors declare no conflicts of interest.

## Supporting information




**Data S1**: Supporting Information.

## Figures and Tables

**FIGURE A1:**
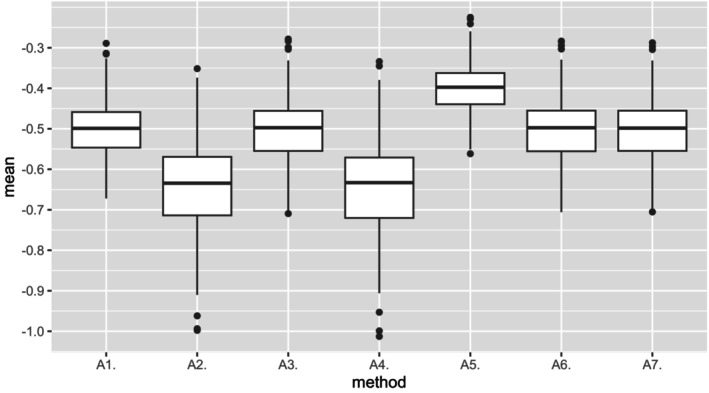
Simulation results. We simulated 500 datasets of size n=1000 with true θ=−0.5 and applied each of the six methods (A2–A7) recording the posterior mean estimate. We also analyzed data before introducing missingness (i.e., from the oracle) and fit the univariate normal model to these data. For reference: A1. = Oracle with univariate Normal model; A2. = Complete‐case analysis with univariate Normal model; A3. = Gelman's 3 step with SVL imputation and univariate Normal model; A4. = Gelman's 3 step with DVL imputation with univariate Normal model; A5. = Gelman's 3 step with JAV imputation with univariate Normal model; A6. = Bivariate Normal model (with mathematics); and A7. = Bivariate Normal model (with MC approx.).

## Data Availability

The data that support the findings of this study are openly available in Bayesian_Missing_Derived_Outcomes at https://github.com/harlanhappydog/Fully_Bayesian_Missing_Derived_Outcomes.git.

## Appendix A

### Simulation Study

Let us now review the results of a small simulation study to confirm that our intuitions regarding each of the possible strategies. We simulated 500 datasets of size n=1000 and applied each of the six methods. The aim was to demonstrate a proof‐of‐concept for our proposed method.

Five hundred complete‐data observations were generated for group A and another five hundred complete‐data observations were generated for group B as follows. For i in 1,…,n

$$\begin{array}{lll} \left(\begin{array}{c} Z_{1}\\ Z_{2} \end{array}\right) & ∼\text{Normal}\left(\begin{array}{c} \left(\begin{array}{c} 1\\ 2 \end{array}\right),\left(\begin{array}{cc} 1 & 0.25\\ 0.25 & 1 \end{array}\right) \end{array}\right),\; & \\ \;\; & \text{if}\;i\;\text{is in group}\;A\; & \\ \left(\begin{array}{c} Z_{1}\\ Z_{2} \end{array}\right) & ∼\text{Normal}\left(\begin{array}{c} \left(\begin{array}{c} 1.5\\ 1 \end{array}\right),\left(\begin{array}{cc} 1 & 0.25\\ 0.25 & 1 \end{array}\right) \end{array}\right),\; & \\ \;\; & \text{if}\;i\;\text{is in group}\;B.\; & \end{array}$$

As in the illustrative example, the derived outcome variable is $Y=Z_{1}+Z_{2}$ and the interest is in estimating θ, the difference between the expectation of Y in group A and in group B. The true value of θ=−0.5.

We analyzed the data prior to introduction of any missingness (i.e., from the “oracle”) with the univariate normal model (see (1)). Missingness was then imposed under a MAR mechanism as follows. All data remained fully observed for group B. For group A, a randomly selected 250 observations had non‐zero probability of $z_{1}$ being missing, while the other 250 had non‐zero probability of $z_{2}$ being missing. The following models determined the probability of missingness in these two subsets of the group A data:

$$\begin{array}{ll} \text{logit}[P(z_{1}\;\text{is missing})] & =10+10z_{2},\\ \text{and logit}[P(z_{2}\;\text{is missing})] & =4-5z_{1}. \end{array}$$

For analysis of the incomplete data, each of the MICE methods was used to produce 50 imputed datasets, each Bayesian model was fit using MCMC with M=5000 draws, and the proposed method was implemented with S=50000 Monte Carlo samples for the integration.

Figure A1 plots the simulation study results. In summary, the we see that:

- The complete‐case analysis (A2) and the three‐step approach with DVL imputation (A4) are both biased as expected, since the data generating mechanism is not MCAR.
- The three‐step approach with SVL imputation (A3) is unbiased.
- Both approaches that fit the bivariate Normal model (A6 and A7) are unbiased and just as efficient as the three‐step approach with SVL imputation (A3).
- Finally, the three‐step approach with JAV (A5) imputation appears to be biased. This is surprising, since in this case JAV imputation should be identical to SVL imputation. A brief explanation of this claim is given in the Appendix. In short, this is not a property of JAV imputation but of the R package {mice} used to implement it.

Recall that JAV imputation used a fully conditional specification procedure – specifically the chained equations flavor – rather than a joint model. Denote a random draw with  and the cth cycle using the superscript . The procedure (typically) initiates in a monotone fashion, visiting variables from least to most missing data, omitting incomplete variables that have not yet been imputed at the zeroth cycle. By construction, Y always has more missing data than $Z_{1}$ or $Z_{2}$ and, in a given repetition, either $Z_{1}$ or $Z_{1}$ may have the least. For illustration, suppose that $Z_{1}$ has the least missing data. The procedure then draws

$$\begin{array}{ll} Z_{1}^{*(0)} & |\text{Group},\\ Z_{2}^{*(0)} & |Z_{1}^{*(0)},Z_{1}^{\left(obs\right)},\text{Group}. \end{array}$$

So far, this is identical to SVL imputation. Next, the procedure aims to draw

$$\begin{array}{ll} Y^{*(0)} & |Z_{1}^{*(0)},Z_{1}^{\left(obs\right)},Z_{2}^{*(0)},Z_{2}^{\left(obs\right)},\text{Group}. \end{array}$$

To do so, it starts by fitting a linear regression model for Y conditioning on $Z_{1}^{\left(obs\right)},Z_{2}^{\left(obs\right)},\text{Group}$ (note that $Z_{1}^{*(1)},Z_{2}^{*(1)}$ are not conditioned‐on because chained equations fits the imputation model for a given variable among those with that variable observed, which makes it more efficient than a Gibbs sampler). Now that Y conditions on the variables that perfectly predict its value, the linear regression model will correctly estimate the coefficients, without uncertainty and without residual error. Even if it draws $Y^{*(0)}$ then $Z_{1}^{*(1)},Z_{1}^{*(1)}$ and so on, the final imputations have already been completely determined by $Z_{1}^{*(0)}$ and $Z_{2}^{*(0)}$. Thus, the procedure should be identical to SVL imputation. Where does the apparent bias come from? We believe it is due to default choices made by the R package mice (possibly to handle perfect prediction, or drop variables; we are unsure). Indeed, to check this, we reran the simulation study using Stata's mi impute chained to perform the imputation and, as expected, found that the JAV procedure described above was unbiased as expected.

### Mathematical Derivation for Dutch Boys Example

Note that:

(A1)
$$\begin{array}{ll} Y & =\log\left(\frac{Z_{2}}{{\left(Z_{1}/100\right)}^{2}}\right)\\ & =\log(Z_{2})-2\log(Z_{1})+2\log(100), \end{array}$$

so that, for the bivariate model we have:

(A2)
$$\begin{array}{ll} Y∼ & \text{Normal}(\gamma_{0}+\gamma_{1}r+\gamma_{2}a+\gamma_{3}ra\\ & -2(\alpha_{0}+\alpha_{1}r+\alpha_{2}a+\alpha_{3}ra)+2\log(100),\\ & \;\;\tau_{Z2}^{2}+4\tau_{Z1}^{2}+2\tau_{Z2}\tau_{Z1}\rho ), \end{array}$$

and:

(A3)
$$\begin{array}{ll} \theta & =\int_{a}E(Y|R=1,A=a)f_{A}(a)\text{da}\\ & \;-\int_{a}E(Y|R=0,A=a)f_{A}(a)\text{da}\\ & =\int_{a}\gamma_{0}+\gamma_{1}1+\gamma_{2}a+\gamma_{3}1a+2\log(100)\\ & \;-2(\alpha_{0}+\alpha_{1}1+\alpha_{2}a+\alpha_{3}1a)\\ & \;-(\gamma_{0}+\gamma_{2}a+2\log(100)-2(\alpha_{0}+\alpha_{2}a))\text{da}\\ & =\int_{a}(\gamma_{1}+\gamma_{3}a-2\alpha_{1}-2\alpha_{3}a)f_{A}(a)\text{da}\\ & =\gamma_{1}-2\alpha_{1}+(\gamma_{3}-2\alpha_{3})E(A). \end{array}$$
