# Supplementary material for: A Fully‐Integrated Bayesian Approach for the Imputation and Analysis of Derived Outcome Variables With Missingness
Source: Stat Med. 2026 Jan 22;45(1-2):e70383. doi: 10.1002/sim.70383 (PMC12826355; doi:10.1002/sim.70383)
Supplement: Supplementary file 1 — Data S1: Supporting Information. [file SIM-45-0-s001.pdf]

if it draws $Y^{*(0)}$ then $Z_1^{*(1)}, Z_1^{*(1)}$ and so on, the final imputations have already been completely determined by $Z_1^{*(0)}$ and $Z_2^{*(0)}$. Thus, the procedure should be identical to SVL imputation. Where does the apparent bias come from? We believe it is due to default choices made by the R package `mice` (possibly to handle perfect prediction, or drop variables; we are unsure). Indeed, to check this, we re-ran the simulation study using Stata's `mi impute chained` to perform the imputation and, as expected, found that the JAV procedure described above was unbiased as expected.

Mathematical derivation for Dutch boys example

Note that:

$$\begin{aligned} Y &= \log\Big(\frac{Z_2}{(Z_1/100)^2}\Big) \\ &= \log(Z_2) - 2\log(Z_1) + 2\log(100), \end{aligned} \tag{19}$$

so that, for the bivariate model we have:

$$\begin{aligned} Y \sim \text{Normal}(&\gamma_0 + \gamma_1 r + \gamma_2 a + \gamma_3 ra \\ &-2(\alpha_0 + \alpha_1 r + \alpha_2 a + \alpha_3 ra) + 2\log(100), \\ &\tau_{Z2}^2 + 4\tau_{Z1}^2 + 2\tau_{Z2}\tau_{Z1}\rho), \end{aligned} \tag{20}$$

and:

$$\begin{aligned} \theta &= \int_a \mathrm{E}(Y|R=1, A=a) f_A(a)\mathrm{da} - \int_a \mathrm{E}(Y|R=0, A=a) f_A(a)\mathrm{da} \\ &= \int_a \gamma_0 + \gamma_1 1 + \gamma_2 a + \gamma_3 1a + 2\log(100) - 2(\alpha_0 + \alpha_1 1 + \alpha_2 a + \alpha_3 1a) - \\ &\quad (\gamma_0 + \gamma_2 a + 2\log(100) - 2(\alpha_0 + \alpha_2 a))\mathrm{da} \\ &= \int_a (\gamma_1 + \gamma_3 a - 2\alpha_1 - 2\alpha_3 a) f_A(a)\mathrm{da} \\ &= \gamma_1 - 2\alpha_1 + (\gamma_3 - 2\alpha_3)\mathrm{E}(A). \end{aligned} \tag{21}$$

SUPPLEMENTAL MATERIAL

R code for the Dutch boys example

```
library(mice)
packageVersion("mice")
# [1] '3.18.0'

library(rjags)
library(mvtnorm)

data(boys)

summary(boys$age)
dim(boys)
boys<-boys[boys[,"age"]>=1 & boys[,"age"]<=18,]


summary(boys$age)
boys$logbmi <- log(boys$bmi)
boys$loghgt <- log(boys$hgt)
boys$logwgt <- log(boys$wgt)
boys$city <- as.numeric(boys$reg=="city")
boys<-(boys[,c("hgt", "wgt", "logbmi", "loghgt", "logwgt", "city", "age")])
```

```
##############################
# Venn diagram
library(ggvenn)
ggvenn(as.data.frame(is.na(boys[,c("logbmi","loghgt","logwgt","city")])), fill_color=c("#0073C2FF", "#EFC000FF", "#868686FF", "#CD534CFF"))

##############################
# Frequentist analysis:
plot(logbmi~age,data=boys[boys[,"age"]>1,], col=boys[boys[,"age"]>1,"city"]+1, pch=20)
mod1 <- lm(logbmi~city*age + I(age^2) ,data=boys[boys[,"age"]>1,])
freq_complete <- round(c(coef(mod1)["city"], confint(mod1)["city",]),3)
freq_complete
summary(mod1)

##############################
# Three-panel dotplot
par(mfrow=c(3,1),
oma = c(2,3,0,0) + 0.1,
          mar = c(1,0,1,0) + 0.8)
plot(logwgt ~age,data=na.omit(boys),  col=rgb(0.5,0.9*city,0.7,alpha=0.5), pch=20, ylab="log(weight)"); mtext("log-weight",adj=0, padj=-0.5)
mod1 <- lm(logwgt ~city*age + I(age^2)  ,data=boys)
summary(mod1)
lines(seq(1,18,0.5),predict(mod1, newdata=data.frame(city=0,age=seq(1,18,0.5))), col=rgb(0.5,0,0.7,alpha=1), lwd=3)
lines(seq(1,18,0.5),predict(mod1, newdata=data.frame(city=1,age=seq(1,18,0.5))), col=rgb(0.5,0.9*1,0.7,alpha=1), lwd=3)

plot(loghgt ~age,data=na.omit(boys), col=rgb(0.5,0.9*city,0.7,alpha=0.5), pch=20, ylab="log(height)"); mtext("log-height",adj=0, padj=-0.5)
mod1 <- lm(loghgt ~city*age + I(age^2)   ,data=boys)
summary(mod1)
lines(seq(1,18,0.5),predict(mod1, newdata=data.frame(city=0,age=seq(1,18,0.5))), col=rgb(0.5,0,0.7,alpha=1), lwd=3)
lines(seq(1,18,0.5),predict(mod1, newdata=data.frame(city=1,age=seq(1,18,0.5))), col=rgb(0.5,0.9*1,0.7,alpha=1), lwd=3)

plot(logbmi~age,data=na.omit(boys), col=rgb(0.5,0.9*city,0.7,alpha=0.5), pch=20)
mod1 <- lm(logbmi ~city*age + I(age^2)  ,data=boys); mtext("log-BMI",adj=0, padj=-0.5)
summary(mod1)
lines(seq(1,18,0.5),predict(mod1, newdata=data.frame(city=0,age=seq(1,18,0.5))), col=rgb(0.5,0,0.7,alpha=1), lwd=3)
lines(seq(1,18,0.5),predict(mod1, newdata=data.frame(city=1,age=seq(1,18,0.5))), col=rgb(0.5,0.9*1,0.7,alpha=1), lwd=3)

title(xlab = "Age (years)",
      ylab = "",
      outer = TRUE, line = 1, cex.lab=1.2)

##############################
nMCMC <- 2000
thedata <- boys[,c("logbmi", "hgt", "wgt", "city", "age")]
dim(thedata)


# Univariate analysis:
# model 1: a model with y is input
jags_univ <- "model {
# Priors
beta0 ~ dnorm(0,1)
beta1 ~ dnorm(0,1)
beta2 ~ dnorm(0,1)
beta3 ~ dnorm(0,1)
beta4 ~ dnorm(0,1)
sigma ~ dexp(1)

# Model
for(i in 1:N){
y[i]     ~ dnorm(beta0 + beta1*x1[i] +
beta2*x2[i]+ beta3*x1[i]*x2[i] + beta4*(x2[i]^2),
 1/(sigma_squared))}

# Output
sigma_squared <- sigma^2
}"




# Bivariate analysis:
# model 2: a model with z1 and z2 as input
jags_biv<- "model {
# Priors
```

```
alpha0 ~ dnorm(0,1)
alpha1 ~ dnorm(0,1)
alpha2 ~ dnorm(0,1)
alpha3 ~ dnorm(0,1)
alpha4 ~ dnorm(0,1)

gamma0 ~ dnorm(0,1)
gamma1 ~ dnorm(0,1)
gamma2 ~ dnorm(0,1)
gamma3 ~ dnorm(0,1)
gamma4 ~ dnorm(0,1)

 # Constructing the covariance matrix and the corresponding precision matrix.
    prec[1:2,1:2] <- inverse(cov[,])
    cov[1,1] <- sigma[1] * sigma[1]
    cov[1,2] <- sigma[1] * sigma[2] * rho
    cov[2,1] <- sigma[1] * sigma[2] * rho
    cov[2,2] <- sigma[2] * sigma[2]

    # Flat priors on all parameters which could, of course, be made more informative.
    sigma[1] ~ dexp(1)
    sigma[2] ~ dexp(1)
    rho ~ dunif(-1, 1)
    pi  ~ dunif(0, 1)
# Model
for(i in 1:N){
  x1[i]  ~ dbin(pi,1);
  mu[i,1] = alpha0 + alpha1*x1[i] +
   alpha2*x2[i] + alpha3*x1[i]*x2[i] + alpha4*(x2[i]^2) ;
  mu[i,2] = gamma0 + gamma1*x1[i] +
   gamma2*x2[i] + gamma3*x1[i]*x2[i] + gamma4*(x2[i]^2);
#z[i,1:2] ~ dmnorm(mu[i,1:2], prec[1:2,1:2]);


z[i,1] ~ dmnorm(mu[i,1], pow(sigma[1],-2) );
z[i,2] ~ dmnorm(mu[i,2] + (sigma[2]/sigma[1])*rho*(z[i,1]-mu[i,1]),
                1/((1-(rho^2))*(sigma[2]^2)) );

}

}"

dim(thedata)

############################################################
############################################################
### ### With univariate ### ###
start_time <- Sys.time()
# model with y as input
thedata_cc<-na.omit(thedata)
jags.m <- jags.model(textConnection(jags_univ),
                     data = list(y = thedata_cc[,"logbmi"],
                                 x1 = thedata_cc[,"city"],
                                 x2 = thedata_cc[,"age"],
                                 N = dim(thedata_cc)[1]))

# this is our estimate
mAsamples <-(coda.samples(jags.m, c("beta1", "beta3") , n.iter = nMCMC, n.burnin=1000))
theta_samples <- apply(mAsamples[[1]],1, function(q) {q["beta1"] + q["beta3"]*mean(thedata[,"age"])})

univariate_complete_est <- round(quantile(theta_samples, c(0.5,0.025,0.975)),3)
end_time <- Sys.time()
mAtime <- end_time-start_time
univariate_complete_time <-mAtime
univariate_complete_est
univariate_complete_time
############################################################
############################################################

####################
create_theta_gcomp <- function(q){

# N <- dim(thedata)[1]
N <- 1
```

```
x1 = rep(0,S*N)
x2 = sample(thedata[,"age"], S*N, replace=TRUE)

mu1 = q["alpha0"] + q["alpha1"]*x1 + q["alpha2"]*x2 + q["alpha3"]*x1*x2 + q["alpha4"]*(x2^2);
mu2 = q["gamma0"] + q["gamma1"]*x1 + q["gamma2"]*x2 + q["gamma3"]*x1*x2 + q["gamma4"]*(x2^2);

Sigma = matrix(c(q["sigma[1]"]^2,
q["sigma[1]"]*q["sigma[2]"]*q["rho"],
q["sigma[1]"]*q["sigma[2]"]*q["rho"],
q["sigma[2]"]^2),2,2)


logBMIstar0 <- apply(cbind(1:(S*N)),1, function(i){
zstar_i <- rmvnorm(1,cbind(mu1[i], mu2[i]), sigma= Sigma)
logBMIstar <- (zstar_i[2]) - log((exp(zstar_i[1])/100)^2)
return(logBMIstar)})

# Y* ~ Y|X1=0,X2=samplex2

x1 = rep(1,S*N)

mu1 = q["alpha0"] + q["alpha1"]*x1 + q["alpha2"]*x2 + q["alpha3"]*x1*x2 + q["alpha4"]*(x2^2);
mu2 = q["gamma0"] + q["gamma1"]*x1 + q["gamma2"]*x2 + q["gamma3"]*x1*x2 + q["gamma4"]*(x2^2);


logBMIstar1 <- apply(cbind(1:(S*N)),1, function(i){
zstar_i <- rmvnorm(1,cbind(mu1[i], mu2[i]), sigma= Sigma)
logBMIstar <- (zstar_i[2]) - log((exp(zstar_i[1])/100)^2)
return(logBMIstar)})
# Y* ~ Y|X1=1,X2=samplex2

thetasample <- mean(logBMIstar1)  -mean(logBMIstar0)

return(thetasample)}
########################################


############################################################
### ### With bivariate and math and proposed ### ###
start_time <- Sys.time()
# model with z as input
jags.m <- jags.model(textConnection(jags_biv),
                     data = list(z = cbind(log(thedata[,"hgt"]),
                                           log(thedata[,"wgt"])),
                                 x1 = thedata[,"city"],
                                 x2 = thedata[,"age"],
                                 N = dim(thedata)[1]))

mAsamples <- coda.samples(jags.m,
c("alpha0", "alpha1", "alpha2", "alpha3", "alpha4",
"gamma0", "gamma1", "gamma2", "gamma3", "gamma4", "sigma", "rho"),
n.iter = nMCMC, n.burnin=1000)
dim(mAsamples[[1]])[1]
end_time <- Sys.time()
biv_time <- end_time-start_time

#library(devtools)
#install_github("psolymos/pbapply")
library(pbapply)

# with math:
start_time <- Sys.time()
theta_samplesA <- apply(mAsamples[[1]],1, function(q) {q["gamma1"] - 2*q["alpha1"] +(q["gamma3"]-2*q["alpha3"])*mean(thedata[,"age"])})
math_est <- round(quantile(theta_samplesA, c(0.5,0.025,0.975)),3)
end_time <- Sys.time()
math_time <- (end_time-start_time) + biv_time


# with gcomp:
start_time <- Sys.time()
x1 = thedata[,"city"]; x2 = thedata[,"age"]; S <- 2000
theta_samples <- pbapply(mAsamples[[1]], 1, create_theta_gcomp)
gcomp_est <- round(quantile(theta_samples, c(0.5,0.025,0.975)),3)
end_time <- Sys.time()
```

```
gcomp_time <- (end_time-start_time) + biv_time





#########################
# Passive imputation strategy:
boys[,c("agecity")] <-(boys[,c("city")]*boys[,c("age")])
boys[,c("loghgt")] <- log(boys[,c("hgt")])
boys[,c("logwgt")] <- log(boys[,c("wgt")])
boys[,c("age_squared")] <- (boys[,c("age")])^2

thedata <- (boys[,c("logbmi", "loghgt", "logwgt", "city", "age", "age_squared", "agecity")])
dim(thedata)
start_time <- Sys.time()
dat <- thedata
init = mice(dat, maxit=0)
head(dat)
meth = init$method
predM = init$predictorMatrix
meth
meth[c("loghgt")]="norm"
meth[c("logwgt")]="norm"
meth[c("age")]="norm"
meth[c("city")]="pmm"
meth[c("logbmi")]="~I(log(exp(logwgt)/((exp(loghgt)/100)^2)))"
meth[c("age_squared")]="~I(age^2)"
meth[c("agecity")]="~I(age*city)"
meth
n_imputations <- 50
imputed = mice(dat, method=meth, predictorMatrix=predM, m=n_imputations)
imputed_dat <- list()
for(j in 1:n_imputations){imputed_dat[[j]] <- complete(imputed,action=j)}

coda_samples <- NULL
for(j in 1:n_imputations){

  jags.m1 <- jags.model(textConnection(jags_univ),
                       data = list(y = imputed_dat[[j]][,"logbmi"],
                                   x1 = imputed_dat[[j]][,"city"],
                                   x2 = imputed_dat[[j]][,"age"],
                                   N = dim(imputed_dat[[j]])[1]))




  mAsamples <-(coda.samples(jags.m1, c("beta1", "beta3") , n.iter = nMCMC, n.burnin=1000))
theta_samples <- apply(mAsamples[[1]],1, function(q) {q["beta1"] + q["beta3"]*mean(imputed_dat[[j]][,"age"])})


  coda_samples <- c(coda_samples, theta_samples)
}
passive_samples <- coda_samples
c(mean(passive_samples), sd(passive_samples))
passive_est <- round(quantile(passive_samples, c(0.500,0.025,0.975)),3)
end_time <- Sys.time()
passive_time <- end_time-start_time
passive_est

univariate_complete_est
as.numeric(univariate_complete_time[1])/60

passive_est
as.numeric(passive_time[1])/60

math_est
as.numeric(math_time[1])/60

gcomp_est
as.numeric(gcomp_time[1])/60
```

```
library(ggplot2)
library(dplyr)

# Example data (replace with your actual estimates & CIs)
df <- data.frame(
  dataset = c("Univariate models", "Univariate models",
              "Bivariate models", "Bivariate models"),
  method = c("1. Complete case data", "2. Full data (on-the-fly MI)",
             "3. Mathematics", "4. Proposed method"),
  mean = c(univariate_complete_est[1],
      passive_est[1],
           math_est[1],
           gcomp_est[1]),
  lower = c(univariate_complete_est[2],
      passive_est[2],
           math_est[2],
           gcomp_est[2]),
  upper = c(univariate_complete_est[3],
      passive_est[3],
           math_est[3],
           gcomp_est[3]),
  time_mins = c(
  paste(round(as.numeric(univariate_complete_time[1])/60,2)),
  paste(round(as.numeric(passive_time[1])/60,2),"mins"),
  paste(round(as.numeric(math_time[1])/60,2),"mins"),
  paste(round(as.numeric(gcomp_time[1])/60,2),"mins"))
)

# Factor for ordering in the plot (reverse so top-to-bottom matches)
df <- df %>%
  mutate(method_label = factor(method,
                               levels = rev(unique(method))),
         dataset=  factor(dataset,
                               levels = c("Univariate models",
                               "Bivariate models"))
                               )

# Create plot
ggplot(df, aes(x = mean , y = method_label)) +
  geom_errorbarh(aes(xmin = lower, xmax = upper), height = 0.25, color = "grey50") +
  geom_point(size = 3, color = "grey20") +
  geom_text(aes(label = paste0(time_mins), x = upper + 0.005),
            hjust = 0, size = 3.2) +
  facet_wrap(~dataset, ncol = 1, scales = "free_y") +
  scale_x_continuous(name = "Difference in logBMI", limits = c(-0.025, 0.1)) +
  theme_minimal(base_size = 12) +
  theme(panel.grid.major.y = element_blank(),
        panel.grid.minor = element_blank(),
        strip.text = element_text(face = "bold"),
        axis.title.y = element_blank(),
        axis.title = element_text(size = 10))
```

R code for the ZIKV example

```
### load libraries:

library(rstan)
#options(mc.cores = parallel::detectCores())
library(growthstandards)
library(sn)
library(bayesplot)
library(ggplot2)
library(devtools)
#devtools::install_github("kassambara/ggpubr")
library(ggpubr)
getwd()
###  Data from Villar et al. (2014) - Figure 2C
villar_plot_male <- read.csv("~/Documents/plot-data-male.csv")
villar_plot_female <- read.csv("~/Documents/plot-data-female.csv")

villar <- rbind(cbind(villar_plot_male,sex=0),
```

```
cbind(villar_plot_female,sex=1))
summary(lm(y~sex+x+I(x^2)+ sex:x+  sex:I(x^2),data= villar))

par(mar=c(4,4,4,4))
plot(x= villar$x,y=(villar$y), col=villar$sex+1, pch=20,
ylab="Head circumference (cm)",xlab="Gestational age (weeks)")

round(coef(lm(y~I(x-39)+sex+I(I(x-39)^2),data= villar)),3)

lines(x=c(c(33.5, 34:42, 42.5)),
y=(coef(lm(y~x+sex+I(x^2),data= villar))[c(1,2,4)])%*%t(cbind(1,
c(33.5, 34:42, 42.5), c(c(33.5, 34:42, 42.5))^2)))

lines(x=c(c(33.5, 34:42, 42.5)),
y=(coef(lm(y~x+sex+I(x^2),data= villar))[c(1,2,3,4)])%*%t(cbind(1,
c(33.5, 34:42, 42.5),1, c(c(33.5, 34:42, 42.5))^2)), col="red")

legend("topleft", c("Male","Female"), col=c("black", "red"), pch=20)
text(38.5,31,  "y = 33.919 - 0.450xSex + 0.399x(GA-39) - 0.016x(GA-39)^2")

###################################################################
# Stan models
###################################################################

Bernoulli_model <-
  "data {
  int<lower=0> N;     // Number of observations
  int<lower=0, upper=1> y[N];   // Binary outcome data
  real<lower=0> a;               // Beta distribution shape parameter
  real<lower=0> b;               // Beta distribution shape parameter
}

parameters {
  real<lower=0, upper=1> theta;  // Probability of success
}

model {
  // Likelihood
  for (i in 1:N) {
    y[i] ~ bernoulli(theta);
  }

  // Priors
  theta ~ beta(a, b);
}"


#https://gist.github.com/rmcelreath/9406643583a8c99304e459e644762f82
BsNmN_model <- "data {
  int<lower=0> N;
  int<lower=0, upper=N> N_z1obs;
  int<lower=0, upper=N> N_z2obs;
  int<lower=0, upper=N> N_z3obs;
  int<lower=0, upper=N> N_z1mis;
  int<lower=0, upper=N> N_z2mis;
  int<lower=0, upper=N> N_z3mis;
  vector<lower=0, upper=1>[N_z1obs] z1_obs;  // sex variable
  vector[N_z2obs] z2_obs;             // gestational age variable
  vector[N_z3obs]  z3_obs;             // head circumf. variable
  int<lower=0, upper=1> z1mis_ind[N];
  int<lower=0, upper=N> ii_z1_obs[N_z1obs];
  int<lower=0, upper=N> ii_z2_obs[N_z2obs];
  int<lower=0, upper=N> ii_z3_obs[N_z3obs];
  int<lower=0, upper=N> ii_z1_mis[N_z1mis];
  int<lower=0, upper=N> ii_z2_mis[N_z2mis];
  int<lower=0, upper=N> ii_z3_mis[N_z3mis];
}

parameters {
  vector<lower=0, upper=1>[N_z1mis] z1_mis;
  vector[N_z2mis]  z2_mis;
  vector[N_z3mis]  z3_mis;
  real<upper=-1> kappa;
  real beta01;
```

```
  vector<lower=0>[2] zeta;
  real beta1;
  real beta2;
  real beta3;
  simplex[2] mixweight;

  real mu; // mean of X
  real<lower=0> sigma; // SD of X
  real omega; // shape of X
}

transformed parameters {
  vector[N] z1;  // sex variable
  vector[N] z2;  // gestational age variable
  vector[N] z3;  // head cir variable
  z1[ii_z1_obs] = z1_obs;
  z1[ii_z1_mis] = z1_mis;
  z2[ii_z2_obs] = z2_obs;
  z2[ii_z2_mis] = z2_mis;
  z3[ii_z3_obs] = z3_obs;
  z3[ii_z3_mis] = z3_mis;
  vector[2] beta0;
  real loc_x; // location of X
  real gm_x; // intermediate calculation for location and scale

  gm_x = sqrt(2/pi())*omega/sqrt(1+omega^2);
  loc_x = mu - sigma*gm_x/sqrt(1-gm_x^2);
  beta0[1] = beta01;
  beta0[2] = kappa;

}
model {
  // Prior distributions for GA
  mu ~ normal(0, 0.1);
  sigma ~ inv_gamma(2, 2);
  omega ~ normal(0, 2);

  // Prior distributions for HC:
  beta0[1] ~ normal(0, 0.1);
  beta0[2] ~ normal(-2, 2)T[,-1];
  zeta[1] ~ inv_gamma(2, 2);
  zeta[2] ~ inv_gamma(2, 2);
  beta1 ~ normal(-0.450, 0.1);
  beta2 ~ normal(0.399, 0.1);
  beta3 ~ normal(-0.016, 0.1);

  // Likelihood
 z1_mis ~ uniform(0,1);

 z2_obs ~ skew_normal(loc_x, sigma, omega);
 z2_mis ~ skew_normal(loc_x, sigma, omega);

 vector[2] log_mixweight = log(mixweight);
 for (n in 1:N) {
  vector[2] lps = log_mixweight;
   if (z1mis_ind[n]==1 ) {

// for unknown sex
    for (k in 1:2) {
  lps[k] += log_mix( z1[n],
                    normal_lpdf( z3[n] | 33.912 + beta0[k] + beta1 + beta2*z2[n] +
                    beta3*pow(z2[n],2), zeta[k]),
                    normal_lpdf( z3[n] | 33.912 + beta0[k]  + beta2*z2[n]+
                    beta3*pow(z2[n],2), zeta[k]));
     }
   }
// for known sex
else {
for (k in 1:2) {
      lps[k] += normal_lpdf(z3[n] | 33.912 + beta0[k] +
       beta1*z1[n] + beta2*z2[n]+
                    beta3*pow(z2[n],2), zeta[k]);
     }
   }
```

```
   target += log_sum_exp(lps);
  }
}
"


BsNmN_prior <- "
parameters {

  real<upper=-1> kappa;
  real beta01;
  vector<lower=0>[2] zeta;
  real beta1;
  real beta2;
  real beta3;
  simplex[2] mixweight;

  real mu; // mean of X
  real<lower=0> sigma; // SD of X
  real omega; // shape of X
}

transformed parameters {
  vector[2] beta0;
  real loc_x; // location of X
  real gm_x; // intermediate calculation for location and scale

gm_x = sqrt(2/pi())*omega/sqrt(1+omega^2);
loc_x = mu - sigma*gm_x/sqrt(1-gm_x^2);

beta0[1] = beta01;
beta0[2] = kappa;
}

model {
  // Prior distributions for GA
  mu ~ normal(0, 0.1);
  sigma ~ inv_gamma(2, 2);
  omega ~ normal(0, 2);

  // Prior distributions for HC:
  beta0[1] ~ normal(0, 0.1);
  beta0[2] ~ normal(-2, 2)T[,-1];
  zeta[1] ~ inv_gamma(2, 2);
  zeta[2] ~ inv_gamma(2, 2);

  beta1 ~ normal(-0.450, 0.1);
  beta2 ~ normal(0.399, 0.1);
  beta3 ~ normal(-0.016, 0.1);
    }

"

Bernoulli_model_stan <- stan_model(model_code = Bernoulli_model)
BsNmN_model_stan <- stan_model(model_code = BsNmN_model)
BsNmN_prior_stan <- stan_model(model_code = BsNmN_prior)


##################
## evaluate properties of the prior
##################

set.seed(1234)

# Sample from the prior distribution
stan_fit <- sampling(BsNmN_prior_stan,
                     pars=c("mu","sigma",
                            "omega", "beta0", "beta1",
                            "beta2", "beta3", "zeta", "mixweight"),
                     data = NULL,
                     chains = 4,
                     iter = 11000,
                     warmup = 1000)
```

```
thedraws <- data.frame(rstan::extract(stan_fit))

M <- dim(thedraws)[1]
S <- 5000
ybar<-rep(NA,M)
zscore_star<-matrix(NA,M,S)

for(m in 1:M){
  if(round(m/100)==(m/100)){print(m/M)}
  z1 <- as.numeric(runif(S)>0.5)

  z2 <- rsn(S, thedraws[m,"mu"] - thedraws[m,"sigma"]*0.7978846*(thedraws[m,"omega"])/sqrt(1+(thedraws[m,"omega"])^2),
            thedraws[m,"sigma"],
            thedraws[m,"omega"])

  z3<-rep(NA,S)
 if(round(thedraws[m,"mixweight.1"]*S)>=1){
z3[1:round(thedraws[m,"mixweight.1"]*S)] <- rnorm(round(thedraws[m,"mixweight.1"]*S), 33.912 +
thedraws[m,"beta0.1"] + thedraws[m,"beta1"]*(z1) + thedraws[m,"beta2"]*z2 + thedraws[m,"beta3"]*(z2^2),
            thedraws[m,"zeta.1"])}


if(round(thedraws[m,"mixweight.1"]*S)<S){
 z3[(1+round(thedraws[m,"mixweight.1"]*S)):S] <- rnorm(length((1+round(thedraws[m,"mixweight.1"]*S)):S), 33.912 +
thedraws[m,"beta0.2"] + thedraws[m,"beta1"]*(z1) + thedraws[m,"beta2"]*z2 + thedraws[m,"beta3"]*(z2^2),
            thedraws[m,"zeta.2"])}


  # 42 and above is 42 for the purposes of calculating the z-score:
  z2[(z2)>3]<-3
  zscore_star[m,1:S] <- igb_hcircm2zscore(gagebrth = ((39+z2)*7),
                                          hcircm=z3,
                                          sex=ifelse(z1== 0, "Male","Female"))

  y_star <- as.numeric(zscore_star[m,1:S]<(-2))
  ybar[m] <- mean(y_star)

}

Q_prior <- 100*round(quantile(na.omit(ybar), seq(0,1,0.1)), 4)
Q_prior

# implied prior on risk of microcephaly:
ybar_prior <- ybar
## determining an "equivalent" Beta prior:
ybarm <- as.numeric(na.omit(ybar_prior))

betaparms <- EnvStats::ebeta(ybarm, method = "mle")
betaparmsn <- round(as.numeric(unlist(betaparms)[c("parameters.shape1", "parameters.shape2")]),2)
betaparmsn

Q_simple_prior <- 100*round(quantile(rbeta(M*100,betaparmsn[1] ,betaparmsn[2]), seq(0,1,0.1)),4)

# compare two priors in terms of quantiles:
round(Q_simple_prior,1)
round(Q_prior,1)

# compare two priors with histograms:
simple_prior <- data.frame(ybar=rbeta(M,betaparmsn[1] ,betaparmsn[2]))
ybardata <- data.frame(ybar=ybar_prior)
zscoreprior <- rbind(cbind(simple_prior,simple="Bernoulli model"),
                     cbind(ybardata,simple="BsNmN model"))
twopriors <- ggplot(data=zscoreprior, aes(x=ybar)) +
  geom_histogram( binwidth=500/M, fill="#69b3a2", color="#e9ecef", alpha=0.9, aes(y = ..density..)) +
  ggtitle("The (implied) prior distribution for the risk of microcephaly") + labs(x="")+
  theme(plot.title = element_text(size=10)) +  xlim(c(0,1)) +  ylim(c(0,10)) + facet_grid(simple~.)

### The histograms plot:
twopriors

### The priorZ plot:
mvalues <- sample(1:M)[1:12]
zscore_starD <- (data.frame(zscore_star)[mvalues,])
dim(zscore_starD)
```

```
zscore_starD[,"draw"] <- 1:dim(zscore_starD)[1]
zscore_star_long <- reshape(zscore_starD, direction="long", v.names = "value",
                            varying = list(1:(dim(zscore_starD)[2]-1)),
                            idvar = "draw", timevar="MCdraw", times=1:(dim(zscore_starD)[2]-1))
head(zscore_star_long)

zscore_star_long_prior <- na.omit(zscore_star_long)

normcurve <- data.frame(x_norm=seq(-15,15,0.1), y_norm=dnorm(seq(-15,15,0.1),0,1))
Zsscorep_prior <- ggplot(data=zscore_star_long_prior, aes(x=value, group=draw, fill=draw)) +
  geom_line(data=normcurve, aes(x=x_norm, y=y_norm, fill=NULL, group=NULL,colour="seagreen"), size=1.5, show.legend=FALSE)+
  ggtitle("Implied distribution of the z-score from 12 random draws from the prior")+
  geom_density(alpha=0,  show.legend = FALSE) +
  geom_vline(xintercept=-2, linetype="dashed")+ylab("density")+
  scale_x_continuous(breaks = seq(-8,8,2), limits=c(-8,8))+theme(plot.title = element_text(size=10))
Zsscorep_prior+labs(x="")



##################
set.seed(1234)
N<-1800
villar_plot_male <- read.csv("~/Documents/plot-data-male.csv")[-c(1,10),]
villar_plot_female <- read.csv("~/Documents/plot-data-female.csv")[-c(1,10),]

villar <- rbind(cbind(villar_plot_male,sex=0),
cbind(villar_plot_female,sex=1))
summary(lm(y~sex + x + I(x^2),data= villar))

coef(lm(y~sex+I(x-39),data= villar))%*%c(1,0,0)

villar_coef <- coef(lm(y~sex+x+I(x^2),data= villar))


z1 <- rbinom(N, 1, 0.5)
z2 <- c(rsn(N, 39 - 3*0.7978846* (-4)/sqrt(1+(-4)^2), 3, -4))
z3 <- rnorm(N, villar_coef[1] + villar_coef[2]*round(z1) +
         villar_coef[3]*(unlist(z2)) + villar_coef[4]*(unlist(z2))^2, 1.16)
z2forcalc<-z2
z2forcalc[(z2forcalc)>42]<-42
zscores <- igb_hcircm2zscore(gagebrth = (z2forcalc*7),
                       hcircm=z3,
                         sex=ifelse(z1==0, "Male","Female"))


truthz2 <- z2
mean(zscores, na.rm=TRUE)
sd(zscores, na.rm=TRUE)

# adding microcephaly cases
micro_cases <- sample(1:length(z3),round(0.12*N))
z3[micro_cases] <- z3[micro_cases]-4

z2forcalc<-z2
z2forcalc[(z2forcalc)>42]<-42
zscores_post <- igb_hcircm2zscore(gagebrth = (z2forcalc*7),
                       hcircm=z3,
                         sex=ifelse(z1==0, "Male","Female"))

true_zscores <- zscores_post
true_micro <- mean(zscores_post<(-2))
true_micro

data_pre <- data.frame(z1,z2,z3)

###################################################
### must seperate into blocks
## a pair of variables are missing depending on the third
##

ilogit<- function(x){exp(x)/(1+exp(x))}
logit<-function(x){log(x/(1-x))}

# adding missingness
```

```
firstblock <- 1:round(length(z2)/2)
secondblock <- (round(length(z2)/2)+1):(length(z2))


missing_z2 <- sample(firstblock, prob=ilogit(-3*scale(z3[firstblock])))[1:(length(firstblock)/4)]

missing_z3 <- sample(secondblock, prob=ilogit(3*scale(z2[secondblock])))[1:(length(secondblock)/4)]

missing_z1 <- sample(c(1:length(z1)),size=round(length(c(1:length(z1)))*0.25))

mean(z3[c(1:length(z3))%in%missing_z2])
mean(z3[!c(1:length(z3))%in%missing_z2])

mean(z2[c(1:length(z3))%in%missing_z3])
mean(z2[!c(1:length(z3))%in%missing_z3])



z1[missing_z1] <- NA
z2[missing_z2] <- NA
z3[missing_z3] <- NA



# assembling the data
data_post <- data.frame(z1,z2,z3)
data_post <- data_post[!(rowSums(is.na(data_post))==3),]
dim(data_post)

look1 <- cbind(data_post,id=1:dim(data_post)[1])

sum(micro_cases %in% look1$id)/length(look1$id)
sum(micro_cases %in% na.omit(look1)$id)/length( na.omit(look1)$id)

length(micro_cases %in% na.omit(look1)$id)

# plotting the data
data_post[,"Sex"] <- factor(data_post[,"z1"], labels=c("Male","Female"))
data_post[,"Z2"] <-(data_post[,"z2"])
data_post[,"Z3"] <-(data_post[,"z3"])

ggplot(data= data_post, aes(x=Z2,y=Z3,
col= Sex))+geom_point()+
labs(x="Gestational age (weeks)", y="Head circumference (cm)")


ggscatterhist(
  na.omit(data_post), x = "Z2", y = "Z3",
  color = "Sex", # comment out this and last line to remove the split by species
  margin.plot = "histogram", # I'd suggest removing this line to get density plots
  margin.params = list(fill = "Sex", color = "black", size = 0.2)
)

z2forcalc<-na.omit(data_post)[,"z2"]
z2forcalc[(z2forcalc)>42]<-42
zscores_me_mis <- igb_hcircm2zscore(gagebrth = (z2forcalc*7),
                       hcircm=na.omit(data_post)[,"z3"],
                         sex=ifelse(na.omit(data_post)[,"z1"]==0, "Male","Female"))

length(zscores_me_mis)
mean(zscores_me_mis<(-2))

##### What data is missing for establishing microcephaly status?
keyvarsNAtable<-NA
keyvarsNAtable <- data.frame(apply(data_post[,c("z1","z2","z3")], 2,function(x) !is.na(unlist(x))))
colnames(keyvarsNAtable)<-c("sex", "gestational age", "head circumference")
colMeans(keyvarsNAtable)

library(ggplot2)
library(ggpolypath)
library(venn);
dd<-venn((keyvarsNAtable),  ilabels = "counts")
venn(keyvarsNAtable, box=FALSE, ggplot=TRUE, ilcs=1.5,
     sncs=0.75, zcolor=c(2,3,4), opacity=0.1, snames=colnames(keyvarsNAtable), ilabels = "counts")
```

```
## zscore plot
# creating very large dataset from identical distribution for plotting "truth"

z1 <- rbinom(N*1000, 1, 0.5)
z2 <- c(rsn(N*1000, 39 - 3*0.7978846* (-4)/sqrt(1+(-4)^2), 3, -4))
z3 <- rnorm(N*1000, villar_coef[1] + villar_coef[2]*round(z1) +
         villar_coef[3]*(unlist(z2)) + villar_coef[4]*(unlist(z2))^2, 1.16)



z2forcalc<-z2
z2forcalc[(z2forcalc)>42]<-42
zscores <- igb_hcircm2zscore(gagebrth = (z2forcalc*7),
                       hcircm=z3,
                         sex=ifelse(z1==0, "Male","Female"))

# adding microcephaly cases
micro_cases <- sample(1:length(z3),round(0.1*N*1000))
z3[micro_cases] <- z3[micro_cases]-4.5

z2forcalc<-z2
z2forcalc[(z2forcalc)>42]<-42
zscores_post <- igb_hcircm2zscore(gagebrth = (z2forcalc*7),
                       hcircm=z3,
                         sex=ifelse(z1==0, "Male","Female"))
truthz2 <- z2
truth_zscores <- zscores_post
truth_micro <- mean(truth_zscores<(-2))
truth_micro
true_micro


#############################################
# Bernoulli model
#############################################

#############################################
### complete case:



# Compile the Stan model
gc()
compiled_Bernoulli_model <- stan_model(model_code = Bernoulli_model)

# Run MCMC sampling
  z1_imp <- na.omit(data_post)[,"z1"]
  z2_imp <- na.omit(data_post)[,"z2"]
  z3_imp <- na.omit(data_post)[,"z3"]

  z2_imp[z2_imp>42]<-42
  zscore_imp <- igb_hcircm2zscore(gagebrth = ((z2_imp)*7),
                                  hcircm=z3_imp,
                                  sex=ifelse(z1_imp==0, "Male","Female"))

  y_imp <- (as.numeric(zscore_imp<(-2)))


# Run MCMC sampling
fit <- sampling(compiled_Bernoulli_model, data = list(a=betaparmsn[1] ,
                                                     b=betaparmsn[2],
                                                     y=y_imp,
                                                     N=length(y_imp)),
                  iter=6000, warmup=1000, chains=1)


completecase_bernoulli <- 100*summary(fit)$summary["theta",c("50%", "2.5%",  "97.5%")]

completecase_bernoulli
truth_micro
#############################################
# Full data
```

```
library(mice)

dat<-data_post
dat$z1<-as.factor(dat$z1)

sum(is.na(dat))
init = mice(dat, maxit=0)
meth = init$method
predM = init$predictorMatrix

meth[c("z1")]="logreg"
meth[c("z2")]="norm"
meth[c("z3")]="norm"
n_imputations<-50
imputed = mice(dat, method=meth, predictorMatrix=predM, m=n_imputations, print = FALSE)
imputed_dat <- list()
compiled_Bernoulli_model <- stan_model(model_code = Bernoulli_model)
for(j in 1:n_imputations){imputed_dat[[j]] <- complete(imputed,action=j)}

coda_samples <- NULL
for(j in 1:n_imputations){
  z1_imp <- imputed_dat[[j]][,"z1"]
  z2_imp <- imputed_dat[[j]][,"z2"]
  z3_imp <- imputed_dat[[j]][,"z3"]

  z2_imp[z2_imp>42]<-42
  zscore_imp <- igb_hcircm2zscore(gagebrth = ((z2_imp)*7),
                                  hcircm=z3_imp,
                                  sex=ifelse(z1_imp==0, "Male","Female"))

  y_imp <- (as.numeric(zscore_imp<(-2)))
  print(table(y_imp,useNA="always"))

  fit <- sampling(compiled_Bernoulli_model, data = list(a=betaparmsn[1] ,
                                                        b=betaparmsn[2],
                                                        y=y_imp,
                                                        N=length(y_imp)),
                  iter=6000, warmup=1000, chains=1)

  coda_samples <- c(coda_samples, c(rstan::extract(fit)$theta))
}
source_samples <- coda_samples
fulldata_bernoulli <- c(100*quantile(source_samples, 0.5),
                        100*quantile(source_samples, c(0.025,0.975)))


#############################################
# Bernoulli-skew-normal model
#############################################

#############################################
### complete case:

naomitdat  <- na.omit(data_post)
N<-dim(naomitdat)[1]
ii_z1_obs <- c(1:N)[!is.na(naomitdat[,"z1"])]
ii_z1_mis <- (c(1:N)[is.na(naomitdat[,"z1"])])
N_z1obs <- length(ii_z1_obs)
N_z1mis <- length(ii_z1_mis)

ii_z2_obs <- c(1:N)[!is.na(naomitdat[,"z2"])]
ii_z2_mis <- (c(1:N)[is.na(naomitdat[,"z2"])])
N_z2obs <- length(ii_z2_obs)
N_z2mis <- length(ii_z2_mis)

ii_z3_obs <- c(1:N)[!is.na(naomitdat[,"z3"])]
ii_z3_mis <- (c(1:N)[is.na(naomitdat[,"z3"])])
N_z3obs <- length(ii_z3_obs)
N_z3mis <- length(ii_z3_mis)

z1mis_ind <- ifelse( 1:N %in% ii_z1_mis , 1 , 0 )

# Create data list for Stan
data_list <- list(N = dim(naomitdat)[1],
```

```
                  N_z1obs=N_z1obs,
                  N_z1mis=N_z1mis,
                  N_z2obs=N_z2obs,
                  N_z2mis=N_z2mis,
                  N_z3obs=N_z3obs,
                  N_z3mis=N_z3mis,
                  ii_z1_obs = ii_z1_obs,
                  ii_z2_obs = ii_z2_obs,
                  ii_z3_obs = ii_z3_obs,
                  ii_z1_mis = ii_z1_mis,
                  ii_z2_mis = ii_z2_mis,
                  ii_z3_mis = ii_z3_mis,
                  z1mis_ind = z1mis_ind,
                  z1_obs = na.omit(naomitdat[,"z1"]),
                  z2_obs = na.omit(naomitdat[,"z2"]-39),
                  z3_obs = na.omit(naomitdat[,"z3"]))


gc()
# Sample from the posterior distribution
init_fun<-function(){list()}
completecase_BsNmN_fit <- sampling(BsNmN_model_stan,
                                             pars=c("mu", "beta0","beta1", "beta2", "beta3",
                                                    "sigma","omega", "zeta", "mixweight"),
                                             data = data_list, chains = 1,
                                             iter = 6000, warmup = 1000,
                                             control=list(max_treedepth=8))


stan_fit <- completecase_BsNmN_fit
summary(stan_fit)
# Diagnostics using bayesplot
# Trace plot
mcmc_trace(stan_fit)
thedraws <- data.frame(rstan::extract(stan_fit))

M <- dim(thedraws)[1]
S <- 5000
ybar<-rep(NA,M)
zscore_star<-matrix(NA,M,S)


for(m in 1:M){
  if(round(m/100)==(m/100)){print(m/M)}
  z1 <- as.numeric(runif(S)>0.5)

  z2 <- rsn(S, thedraws[m,"mu"] - thedraws[m,"sigma"]*0.7978846*(thedraws[m,"omega"])/sqrt(1+(thedraws[m,"omega"])^2),
            thedraws[m,"sigma"],
            thedraws[m,"omega"])


  z3<-rep(NA,S)
 if(round(thedraws[m,"mixweight.1"]*S)>=1){
z3[1:round(thedraws[m,"mixweight.1"]*S)] <- rnorm(round(thedraws[m,"mixweight.1"]*S), 33.912 +
thedraws[m,"beta0.1"] + thedraws[m,"beta1"]*(z1) + thedraws[m,"beta2"]*z2 + thedraws[m,"beta3"]*(z2^2),
            thedraws[m,"zeta.1"])}


if(round(thedraws[m,"mixweight.1"]*S)<S){
 z3[(1+round(thedraws[m,"mixweight.1"]*S)):S] <- rnorm(length((1+round(thedraws[m,"mixweight.1"]*S)):S), 33.912 +
thedraws[m,"beta0.2"] + thedraws[m,"beta1"]*(z1) + thedraws[m,"beta2"]*z2 + thedraws[m,"beta3"]*(z2^2),
            thedraws[m,"zeta.2"])}

  # 42 and above is 42 for the purposes of calculating the z-score:
  z2[(z2)>3]<-3
  zscore_star[m,1:S] <- igb_hcircm2zscore(gagebrth = ((39+z2)*7),
                                          hcircm=z3,
                                          sex=ifelse(z1== 0, "Male","Female"))

  y_star <- as.numeric(zscore_star[m,1:S]<(-2))
  ybar[m] <- mean(y_star)

}
```

```
mvalues <- sample(1:M)[1:12]
zscore_starD <- (data.frame(zscore_star)[mvalues,])
dim(zscore_starD)
zscore_starD[,"draw"] <- 1:dim(zscore_starD)[1]
zscore_star_long <- reshape(zscore_starD, direction="long", v.names = "value",
                            varying = list(1:(dim(zscore_starD)[2]-1)),
                            idvar = "draw", timevar="MCdraw", times=1:(dim(zscore_starD)[2]-1))




dens_truth <- data.frame(x=density((truth_zscores),
        adjust=0.7)$x, y=density((truth_zscores),adjust=1)$y)

normcurve <- data.frame(x_norm=seq(-15,15,0.1), y_norm=dnorm(seq(-15,15,0.1),0,1))
Zsscorep <- ggplot(data=zscore_star_long, aes(x=value, group=draw,  colour="Posterior estimates")) +
  geom_line(data=normcurve,alpha=0.75,
    aes(x=x_norm, y=y_norm, fill=NULL, group=NULL,colour="N(0,1)"), size=1.5, show.legend=FALSE)+
  ggtitle("Implied distribution of the z-score from 12 random draws from the prior")+
  geom_density(alpha=0,  show.legend = FALSE, adjust=2) +
  geom_vline(xintercept=-2, linetype="dashed")+ylab("density")+
  scale_x_continuous(breaks = seq(-6,4,2), limits=c(-6,4))+theme(plot.title = element_text(size=10))
Zsscorep+
 geom_line(data=dens_truth,
    aes(x=x, y=y, fill=NULL, group=NULL,colour="Truth"), size=1.5, alpha=0.75,show.legend=TRUE) +
  theme(legend.title=element_blank())

completecasedata_bernoulliskenormal <- c(100*quantile(na.omit(ybar), 0.5),
                100*quantile(na.omit(ybar), c(0.025,0.975)))


#############################################
# Full data

N<-dim(data_post)[1]
ii_z1_obs <- c(1:N)[!is.na(data_post[,"z1"])]
ii_z1_mis <- (c(1:N)[is.na(data_post[,"z1"])])
N_z1obs <- length(ii_z1_obs)
N_z1mis <- length(ii_z1_mis)

ii_z2_obs <- c(1:N)[!is.na(data_post[,"z2"])]
ii_z2_mis <- (c(1:N)[is.na(data_post[,"z2"])])
N_z2obs <- length(ii_z2_obs)
N_z2mis <- length(ii_z2_mis)

ii_z3_obs <- c(1:N)[!is.na(data_post[,"z3"])]
ii_z3_mis <- (c(1:N)[is.na(data_post[,"z3"])])
N_z3obs <- length(ii_z3_obs)
N_z3mis <- length(ii_z3_mis)

z1mis_ind <- ifelse( 1:N %in% ii_z1_mis , 1 , 0 )

# Create data list for Stan
data_list <- list(N = dim(data_post)[1],
                  N_z1obs=N_z1obs,
                  N_z1mis=N_z1mis,
                  N_z2obs=N_z2obs,
                  N_z2mis=N_z2mis,
                  N_z3obs=N_z3obs,
                  N_z3mis=N_z3mis,
                  ii_z1_obs = ii_z1_obs,
                  ii_z2_obs = ii_z2_obs,
                  ii_z3_obs = ii_z3_obs,
                  ii_z1_mis = ii_z1_mis,
                  ii_z2_mis = ii_z2_mis,
                  ii_z3_mis = ii_z3_mis,
                  z1mis_ind = z1mis_ind,
                  z1_obs = na.omit(data_post[,"z1"]),
                  z2_obs = na.omit(data_post[,"z2"]-39),
                  z3_obs = na.omit(data_post[,"z3"]))

gc()
```

```
# Sample from the posterior distribution
init_fun<-function(){list(mixweight=c(0.5,0.5))}
fulldata_BsNmN_fit <- sampling(BsNmN_model_stan,
                                             pars=c("mu", "beta0","beta1", "beta2","beta3",
                                                    "sigma","omega", "zeta", "mixweight"),
                                             data = data_list, chains = 1,
                                             iter = 6000, warmup = 1000,
                                             control=list(max_treedepth=5), init= init_fun)



stan_fit <- fulldata_BsNmN_fit
summary(stan_fit)
# Diagnostics using bayesplot
# Trace plot
mcmc_trace(stan_fit)
thedraws <- data.frame(rstan::extract(stan_fit))

M <- dim(thedraws)[1]
S <- 5000
ybar<-rep(NA,M)
zscore_star<-matrix(NA,M,S)


for(m in 1:M){
  if(round(m/100)==(m/100)){print(m/M)}
  z1 <- as.numeric(runif(S)>0.5)

  z2 <- rsn(S, thedraws[m,"mu"] -
    thedraws[m,"sigma"]*0.7978846*(thedraws[m,"omega"])/sqrt(1+(thedraws[m,"omega"])^2),
            thedraws[m,"sigma"],
            thedraws[m,"omega"])

  z3<-rep(NA,S)
 if(round(thedraws[m,"mixweight.1"]*S)>=1){
z3[1:round(thedraws[m,"mixweight.1"]*S)] <- rnorm(round(thedraws[m,"mixweight.1"]*S), 33.912 +
thedraws[m,"beta0.1"] + thedraws[m,"beta1"]*(z1) + thedraws[m,"beta2"]*z2 + thedraws[m,"beta3"]*(z2^2),
            thedraws[m,"zeta.1"])}


if(round(thedraws[m,"mixweight.1"]*S)<S){
 z3[(1+round(thedraws[m,"mixweight.1"]*S)):S] <- rnorm(length((1+round(thedraws[m,"mixweight.1"]*S)):S), 33.912 +
thedraws[m,"beta0.2"] + thedraws[m,"beta1"]*(z1) + thedraws[m,"beta2"]*z2 + thedraws[m,"beta3"]*(z2^2),
            thedraws[m,"zeta.2"])}



  # 42 and above is 42 for the purposes of calculating the z-score:
  z2[(z2)>3]<-3
  zscore_star[m,1:S] <- igb_hcircm2zscore(gagebrth = ((39+z2)*7),
                                          hcircm=z3,
                                          sex=ifelse(z1== 0, "Male","Female"))

  y_star <- as.numeric(zscore_star[m,1:S]<(-2))
  ybar[m] <- mean(y_star)

}

fulldata_bernoulliskenormal <- c(100*quantile(na.omit(ybar), 0.5),
                100*quantile(na.omit(ybar), c(0.025,0.975)))

######################################################
### RESULTS
######################################################

round(completecase_bernoulli,2)
round(completecasedata_bernoulliskenormal,2)
round(fulldata_bernoulli,2)
round(fulldata_bernoulliskenormal,2)

###### COMPARE TO THE "TRUTH":
true_micro
truth_micro
```

```
######################################################
### PLOT 1
######################################################

mvalues <- sample(1:M)[1:12]
zscore_starD <- (data.frame(zscore_star)[mvalues,])
dim(zscore_starD)
zscore_starD[,"draw"] <- 1:dim(zscore_starD)[1]
zscore_star_long <- reshape(zscore_starD, direction="long", v.names = "value",
                            varying = list(1:(dim(zscore_starD)[2]-1)),
                            idvar = "draw", timevar="MCdraw", times=1:(dim(zscore_starD)[2]-1))

zscore_fulldata_bernoulliskenormal<-zscore_star_long

mvalues <- sample(1:M)[1:12]
zscore_starD <- (data.frame(zscore_star)[mvalues,])
dim(zscore_starD)
zscore_starD[,"draw"] <- 1:dim(zscore_starD)[1]
zscore_star_long <- reshape(zscore_starD, direction="long", v.names = "value",
                            varying = list(1:(dim(zscore_starD)[2]-1)),
                            idvar = "draw", timevar="MCdraw", times=1:(dim(zscore_starD)[2]-1))

zscore_fulldata_bernoulliskenormal<-zscore_star_long

dens_raw <- data.frame(x_raw=density(na.omit(truth_zscores),adjust=0.7)$x,
    y_raw=density(na.omit(truth_zscores),adjust=1)$y)

normcurve <- data.frame(x_norm=seq(-15,15,0.1), y_norm=dnorm(seq(-15,15,0.1),0,1))

Zsscorep <- ggplot(data=zscore_star_long, aes(x=value, group=draw,  colour="Posterior estimates")) +
  ggtitle("Implied distribution of the z-score from 12 random draws from the posterior")+
  geom_vline(xintercept=-2, linetype="dashed")+ylab("density")+
  geom_line(data=normcurve, aes(x=x_norm, y=y_norm, fill=NULL, group=NULL,colour="Normal(0,1)"), size=1.75, show.legend=FALSE, alpha=0.7)+
  scale_x_continuous(breaks = seq(-6,4,1), limits=c(-6,4))+
  theme(plot.title = element_text(size=10))

Zsscorep+
 geom_line(data=dens_raw, aes(x=x_raw, y=y_raw, fill=NULL, group=NULL,colour="Truth"),
    size=1.75, show.legend=TRUE, alpha=0.7)+
  geom_line(stat="density", show.legend = FALSE, adjust=1, alpha=0.5) +
  theme(legend.title=element_blank())

######################################################
### PLOT 2
######################################################

dens_z2 <- data.frame(x=density(na.omit(truthz2),adjust=0.7)$x, y=density(na.omit(truthz2),adjust=1)$y)

impz2 <-NULL
 for(j in 1:50){
impz2 <- rbind(impz2,
cbind(draw=j,value=(imputed_dat[[j]][,"z2"])))}
  head(impz2)

Z2_plot <- ggplot(data= impz2, aes(x=value, group=draw,  colour="Posterior estimates")) +
  ggtitle("Implied distribution of gestational age from 50 imputation models")+
  geom_line(stat="density", show.legend = FALSE, adjust=1, alpha=0.5) +
  theme(legend.title=element_blank())
Z2_plot  +   geom_line(data= dens_z2, aes(x=x, y=y, fill=NULL, group=NULL,colour="Truth"),
    size=1.75, show.legend=FALSE, alpha=0.7)+
  scale_x_continuous(breaks = seq(30,43,1), limits=c(30,43))
```
